# Natives and non‐natives plants show different responses to elevation and disturbance on the tropical high Andes of Ecuador

**DOI:** 10.1002/ece3.3270

**Published:** 2017-08-30

**Authors:** Verónica Sandoya, Aníbal Pauchard, Lohengrin A. Cavieres

**Affiliations:** ^1^ Escuela de Ciencias Biológicas e Ingeniería Universidad Yachay Tech Urcuquí Ecuador; ^2^ Centre de Recerca Ecològica i Aplicacions Forestals Universitat Autònoma de Barcelona Barcelona España; ^3^ Departamento de Botánica Facultad de Ciencias Naturales y Oceanográficas Universidad de Concepción Concepción Chile; ^4^ Laboratorio de Invasiones Biológicas (LIB) Facultad de Ciencias Forestales Universidad de Concepción Concepción Chile; ^5^ Instituto de Ecología y Biodiversidad‐IEB Santiago Chile

**Keywords:** altitude, bioclimatic origin, disturbance, exotic species, mountain region, richness, road, tropics

## Abstract

The aim was to assess patterns of plant diversity in response to elevation and disturbance in a tropical mountain. The study area was located in north‐central portion of the Eastern Cordillera of the Ecuadorian Andes, on a road from 1,150 m a.s.l. (Osayacu) to 4,000 (Papallacta). Along a mountain road spanning a wide altitudinal gradient, at 20 elevations we sampled three plots: one at the roadside and two perpendicular to the roadside. The relationship between elevation and species richness was assessed using linear and quadratic regressions, the effect of disturbance on species richness was determined by ANCOVA and a *t* test with parameters obtained from quadratic equations. Similarity of species composition among the roadside and sites distant was evaluated with the Chao‐Jaccard and classic Jaccard similarity indices, the distribution of non‐native species according to their origin were analyzed with linear and quadratic regression. The native species showed a linearly monotonic decrease with elevation, whereas non‐natives showed a quadratic distribution. Disturbed areas had the greatest number of non‐native species and lower native species richness, showing also a high floristic similarity; less disturbed areas showed the opposite. The non‐native species of temperate origin were more numerous and showed unimodal elevational distribution, while species of tropical origin were few and decreased linearly with elevation. We conclude that in a tropical highland mountain range, native and non‐native plant species respond differently to elevation: native species exhibit a monotonically linear decrease, and non‐native species show a unimodal trend. Disturbance positively affects non‐native species showing higher richness and fewer species turnover. In addition, the non‐native species are located along of the elevational gradient in relation to their biogeographic origin.

## 1 INTRODUCTION

Mountain habitats cover 5% of the Earth's land‐area, harboring ca. 10,000 vascular plant species (Körner, 1999; Nagy & Grabherr, 2009). Thus, despite their small area, mountain habitats harbor a great diversity of plant species and supply many important ecosystem services including clean water (Körner, 1999). Although mountain habitats are generally assumed to have little impacts from human intervention including minimal effects of non‐native invasive species, compared to fertile plains or urban areas, over these there is growing evidence that many non‐native species have started to colonize mountain regions worldwide (e.g., Alexander et al., 2011; Becker, Dietz, Billeter, Buschmann, & Edwards, 2005; McDougall et al., 2011; Morueta‐Holme et al., 2015; Seipel et al., 2012). Elevational gradients on mountain areas are usually comprised by a series of complex biotic and abiotic changes that affect species distribution (McCain & Grytnes, 2010). For example, it is well known that favorable temperatures for growth decrease with elevation acting as a filter for the richness and abundance of plant species either native and non‐native (e.g., Nogués‐Bravo et al. 2008, Kessler et al. 2011; Marini et al., 2011; Alexander et al., 2011; Seipel et al., 2012). However, the response of native plants to elevation differ from non‐natives: While the non‐natives species typically decline to long elevations where the elevation acts as a filter for species that do not possess the proper adaptations to settle successfully in extreme conditions, native species tend to have their specific niches adaptations along the elevational gradient (Alexander et al., 2011). Few studies have explicitly compared the distribution of both native and no‐native species in communities that have little or a lot of disturbance, and how these communities are structured along the same elevational gradient (Marini et al., 2011).

The large majority of the studies describing the elevational distribution of non‐native species have been conducted in temperate areas creating an important gap for understanding these processes in other biomes such as tropical mountains (e.g., Alexander et al., 2011; Becker et al., 2005; Marini et al., 2011; Pauchard & Alaback, 2004; Seipel et al., 2012; Siniscalco, Barni, & Bacaro, 2011). The few studies conducted in tropical habitats correspond to islands such as Hawaii (Daehler, 2005; Kitayama & Mueller‐Dombois, 1995; Wester & Juvik, 1983), La Réunion (Pauchard et al., 2009), and Canary Islands (Arévalo et al., 2005; Arteaga, Delgado, Otto, Fernández‐Palacios, & Arévalo, 2009). While monotonic decreases with elevation have been reported for non‐native species in temperate areas (e.g., Alexander et al., 2011; Marini et al., 2011; Seipel et al., 2012), hump‐shaped distributions have been found in the tropical islands (Arévalo et al., 2005; Pauchard et al., 2009). For native species, the elevational distribution in tropical areas also seems to obey hump‐shaped distributions (Kessler et al., 2011), while in temperate areas, both monotonical and hump‐shaped distributions have been reported (Grytnes & Vetaas, 2002; Grytnes, Heegaard, & Ihlen, 2006; Kluge, Kessler, & Dunn, 2006, Nogués‐Bravo et al. 2008).

In tropical mountains, the establishment of non‐native species along an elevational gradient depends on the degree of preadaptation and plasticity inherent in species for the abiotic conditions at a given site (Alexander et al., 2011; Haider et al., 2010; Seipel et al., 2012, 2015). A match between the overall climatic conditions on the native range and the elevational location on the introduced range can be expected. Thus, according to their biogeographical origin, non‐native species can occupy different elevations along an elevational gradient in a tropical site, depending on the similarity between the climatic conditions of the native range and the site of introduction (Arévalo et al., 2005; Arteaga et al., 2009; Haider et al., 2010). Thus, based on biogeography, we would predict that species from tropical and subtropical origin, for example, will be present in the lower elevations of a gradient in a tropical Mountain (Arévalo et al., 2005; Arteaga et al., 2009; Daehler, 2005; Wester & Juvik, 1983). On the other hand, patterns of disturbance along an elevation gradient may uniquely shape distribution. It is well known that disturbances facilitate the arrival and dispersal of propagules of non‐native species, which potentially could exhibit invasive features on these fragile ecosystems being a significant threat to the ecosystems services they provide (Alexander, Naylor, Poll, Edwards, & Dietz, 2009; Arévalo et al., 2005; Arteaga et al., 2009; Becker et al., 2005; Parendes & Jones, 2000; Pauchard & Alaback, 2004; Pauchard et al., 2009).

While it has been widely reported that anthropogenic disturbances affect the presence and abundance of non‐native species (Catford et al., 2012; Pollnac, 2012; Rejmánek, 1989), little is known on disturbance gradients in tropical mountains. In the tropics, the land‐use change, the human settlements, and their access to the mountains are associated with disturbances that could be influencing the distribution of non‐native species. Thus, it is important to evaluate how these communities are structured.

To gain insights about the factors affecting the presence and distribution of non‐native species along an elevational gradient in a tropical continental mountain, we analyzed the elevational distribution of both native and non‐native species in the tropical High Andes of Ecuador. To understand the species responses to increasing elevation and disturbance, we compared the elevational distribution of both native and non‐native species away from and at the roadside as indicators of undisturbed and disturbed habitat, respectively. In particular, we assessed: 1) the distributional pattern of native and non‐native species with elevation; 2) the variation in richness and composition of native and non‐native species with respect to disturbance (distance to road) along an elevational gradient; and 3) the distribution of non‐native species along the elevational gradient respective to its biogeographic origin.

## 2 METHODS

### 2.1 Study area

**Figure 1:**
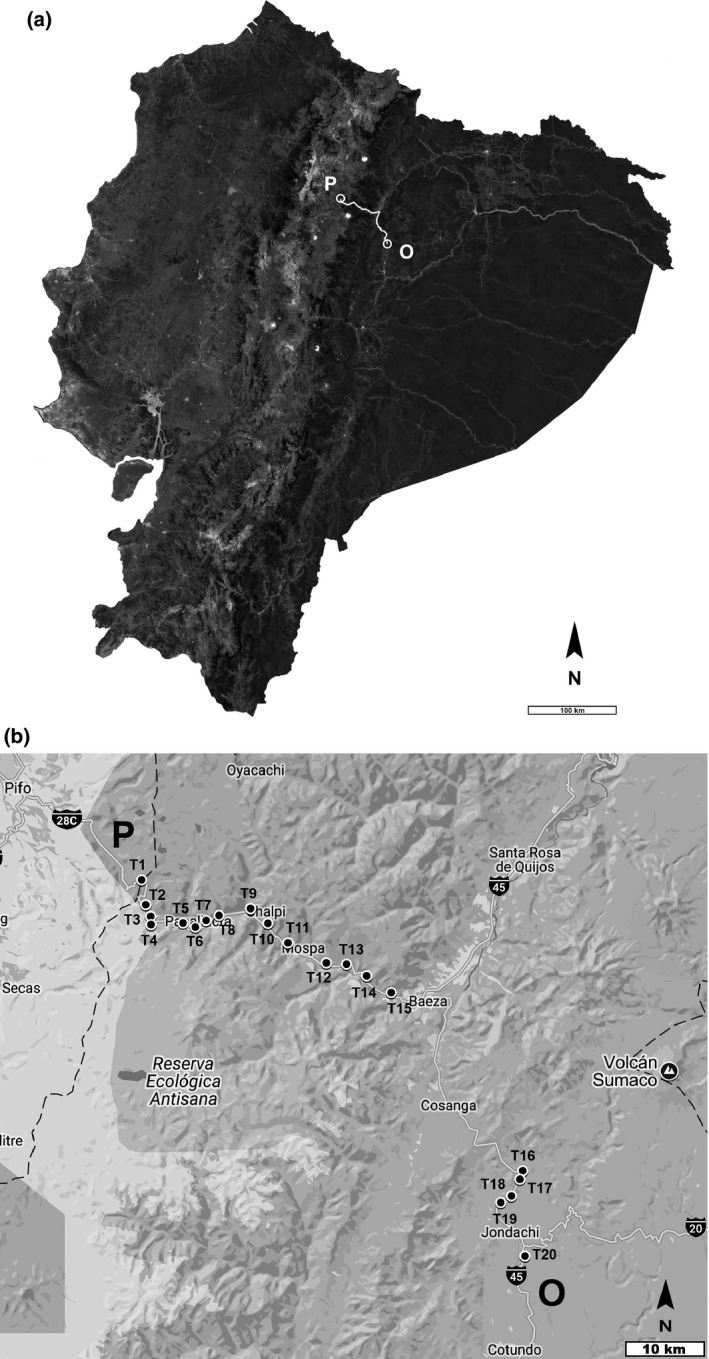
(a) Map of the study area. Road from Paramo Papallacta (P) to Osayacu (O), east of Ecuador. (b) Location of transects (T) along the road, between 4,000 (T1) to 1,150 (T20) m a.s.l. (Adapted to Image © 2017 Google Earth Pro/My Maps)

The study was conducted in the oriental region of the Ecuadorian Andes. We studied the plant communities along a paved road across an elevational range from 1,150 m (0°44′34′′S–77°47′24′′W) to 4,000 (0°19′56′′S–78°12′07′′W) m a.s.l., at 96 km from the Papallacta Paramo (upper limit) to Osayacu (lower limit) (Figure 1a). The study area spans different bioclimatic regions with characteristic vegetation, which vary in structure and composition (Cañadas, 1983; Sierra, 1999) (see Table 1 for details).

**Table 1:** Bioclimatic regions and vegetation types, with their abiotic features, present in the study area

Bioclimatic region	Er (m a.s.l.)	P (mm)	T (°C)	Vegetation types	Er (m a.s.l.)
Wet subtropical	300–1,800	2,000–3,000	23–26	Evergreen premontane forest	800–1,300
Moist temperate	1,800–3,000	1,000–1,500	18	Evergreen lower montane forest	1,300–2,000
				Montane cloud forest	2,000–2,900
Wet subtemperate	3,000–4,000	1,000–1,500	6–12	Evergreen upper montane forest	2,900–3,600
				Páramo grassland	3,500–4,000

T, mean annual temperature.

P, mean annual precipitation.

Er, Elevation range (Cañadas, 1983; Sierra, 1999).

### 2.2 Sampling of vegetation

We used the methodology proposed by MIREN (Mountain Invasions Research Network, see Seipel et al., 2012), designed to evaluate and monitor non‐native invasive species present in mountain ecosystems. Along the elevational gradient, we established 20 transects, located every 150‐m elevation (Figure 1b). The location of transects along the road (left or right) was determined in the field, depending on accessibility for vegetation sampling.

At each transect, three 2 × 50 m^2^ plots were laid out in a T‐shaped transect, so that one plot laid parallel to the road, centered on the sampling location.

The other two plots extended perpendicularly from the road (centered at the sampling location), and midpoints of the plots were 25 and 75 m from the roadside. Each plot was divided into ten 2 × 5 m^2^ subplots where we recorded the presence and cover of native and non‐native species. The intensity of disturbance at each plot was estimated through a visual estimation of loss and/or alteration of vegetation in three categories: (1) undisturbed: structure and composition of vegetation with no visual modification compared to surrounding vegetation, (2) disturbed: the presence of visual alteration in vegetation, and (3) very disturbed, with evident evidence of alterations in the structure and composition of vegetation, with prominent presence of bare soil. Road‐side plots always qualified as very disturbed.

The native or non‐native status of the species recorded on sampling was determined according to the catalog of vascular plants of Ecuador (Jørgensen & León‐Yánez, 1999). Non‐native species were classified according to their biogeographical origin in: temperate (from Palaearctic and Holarctic regions), tropical (from paelotropical and neotropical regions), and cosmopolitan according to Fuentes, Pauchard, Sanchez, Esquivel, and Marticorena (2013). The species collected were identified by specialists, and voucher specimens were deposited in HQCA (Herbarium of the Pontificia Universidad Catolica del Ecuador) and HQCNE (Herbarium of the Natural History Museum of Ecuador). The taxonomy was standardized according to the database to the Missouri Botanical Garden database (Tropicos.org, 2012). The life form of each species was recorded following León‐Yánez et al. (2011).

### 2.3 Data analysis

The relationship between species richness of native and non‐native species with elevation was assessed by fitting linear and quadratic regressions models and according to the R square obtained we selected that with the higher R square. To assess the relationship between the distribution of non‐native species along the elevational gradient with their biogeographic status, we also used linear and quadratic regression models for tropical and temperate origin.

Analysis of covariance (ANCOVA) was used to assess if distance from the road (a proxy of disturbance) affect native species richness across elevations, where native species richness was the dependent variable, distance from the road (roadside, medium‐distance, and distant from the road plots) was the independent variable and elevation as covariate. As the elevational distribution of non‐native species followed a hump‐shaped model (see results), to assess if disturbance affects the elevational distribution of non‐native species, for each distance to the road we obtained the values and standard deviations of the parameters of a quadratic model (*ax² + bx + c*) and were then compared among them with Student's *t* tests. To be rigorous, we considered that there were significant differences among distance to roads when all the quadratic model parameters (*a, b,* and *c*) showed significant differences.

To compare the similarity between the composition of native and non‐native species among the different plots, we used Chao‐Jaccard (based on abundance) and classic Jaccard (presence–absence species) indices of similarity.

All statistical analyzes were performed at 95% confidence level, using the softwares EstimateS 8.20 and SPSS 15.0.

## 3 RESULTS

### 3.1 Total diversity

We found a total of 771 species, where 728 were native species and 43 non‐native species (see Appendix 1). These species belonged to 120 families where the most diverse were as follows: Asteraceae (11%) and Poaceae (7%). The number of genera found was 420, where the most diverse were *Solanum* (21 species), *Miconia* (3%) for native species, and *Trifolium* (7%) for non‐native species. The most abundant native species across the gradient was *Baccharis latifolia* (2%) and the non‐native species *Pennisetum clandestinum* (12%). The non‐native species present in a higher number of transects were as follows: *Conyza bonariensis* (18 transects), *Cerastium glomeratum* (14 transects), *Holcus lanatus* (14 transects), and *Taraxacum officinale* (13 transects).

The herbaceous habit was the most frequent for both native (365 species) and non‐native species (40 species), followed by the shrubs (196 native and three non‐native species).

### 3.2 Elevational distribution patterns

**Figure 2:**
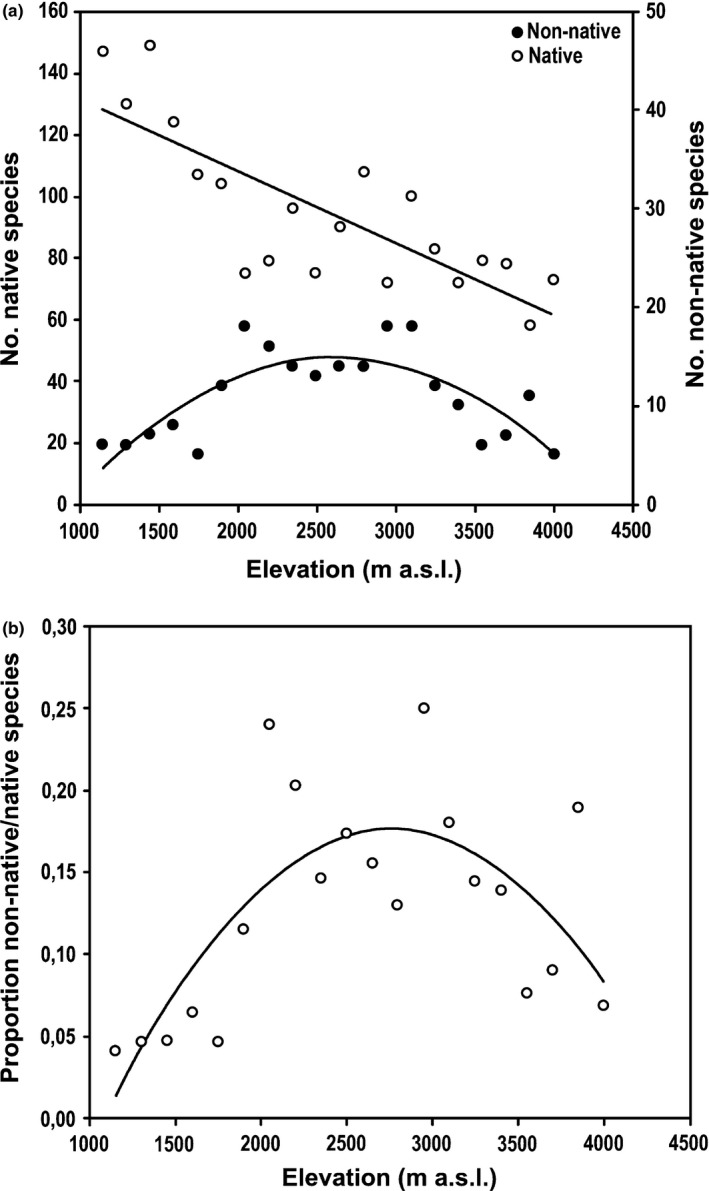
(a) Distribution patterns of native and non‐native species in the T transects along the elevational gradient. (b) Proportion of non‐native and native species along elevational gradient (regression results in Table 2)

Compared with non‐native species, the number of native species was always higher at all elevations. Two clearly defined distribution patterns were found along the gradient for native and non‐native species, respectively (Table 2). For native species, we found a linear monotonic decline (*R*² = 0.62, *p* < .001). In contrast, for non‐native species, we found a unimodal or hump‐shaped pattern (quadratic), with an increase in the number of species in the middle areas of the gradient (*R*² = 0.58, *p* < .001) (Figure 2a). In addition, the proportion of non‐native over native species also showed a quadratic trend (*R*² = 0.46, *p* = .002) (Figure 2b, Table 2).

**Table 2:** Results of regression models with respect species richness, native, and non‐native, with the elevation

Species group	All species			Native species			Non‐native species		
	Model	*R*²	*p*	Model	*R*²	*p*	Model	*R*²	*p*
Along gradient	Linear	0.7	<.001	Linear	0.6	<.001	Quadratic	0.6	<.001
*Roadside*	–	–	–	a	a	a	Quadratic	0.6	<.001
*Near* road	–	–	–	Linear	0.5	<.001	Quadratic	0.4	.01
*Far* road	–	–	–	Linear	0.3	.01	Quadratic	0.3	.03
Non‐native species temperate origin	–	–	–	–	–	–	Quadratic	0.7	<.001
Non‐native species tropical origin	–	–	–	–	–	–	Linear	0.8	<.001
Cosmopolitan species	–	–	–	–	–	–	a	a	a

*R*², *R*² corrected.

*p*,* p* value for the model.

a no tendency.

not applicable.

### 3.3 Disturbance and species distribution

All plots located at the *roadside* contained non‐native species and always showed the highest level of disturbance (80% very disturbed), while the plots located away from the road (*far* plots) showed the least disturbance (80% disturbed and undisturbed 20%). Plots located at an intermediate distance to the road (*near* plots) showed intermediate values of disturbance.

**Figure 3:**
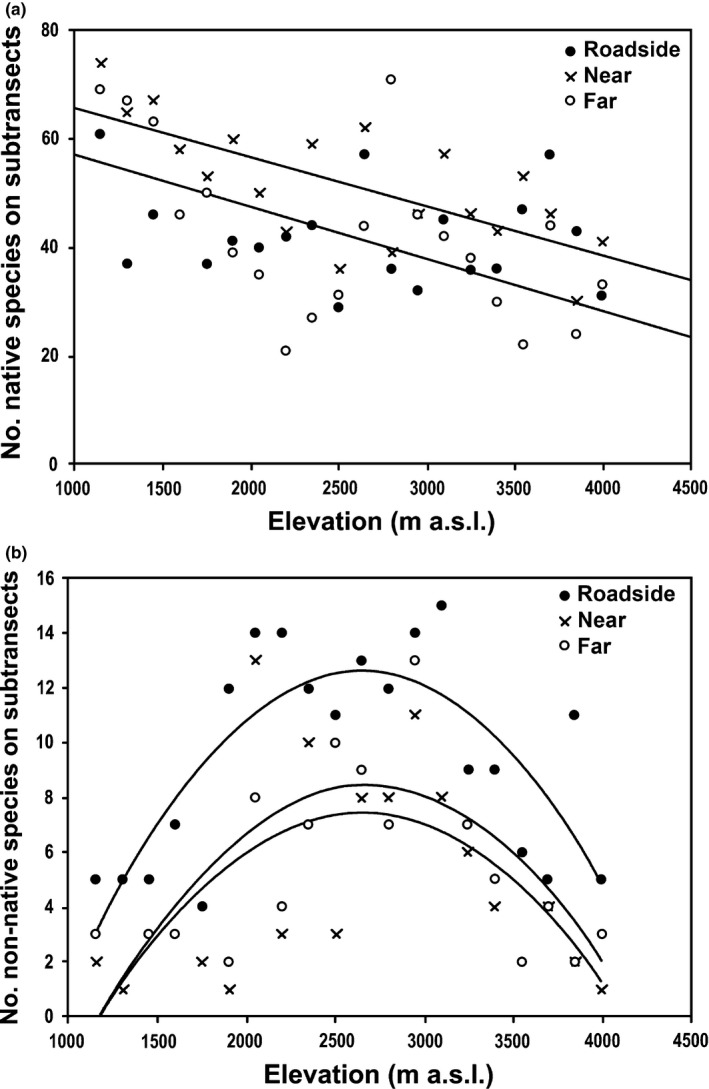
(a) Linear regression for native species on the subtransects (roadside, near, far), along of the elevational gradient. (b) Quadratic regression for non‐native species on subtransects (roadside, near and far), along of the elevational gradient (regression results in Table 2)

Distribution models for native species richness at the different plots were linear (*near*:* R*² = 0.47, *p* < .001; *far*:* R*² = 0.30, *p* = .012) (Figure 3a, Table 2). ANCOVA results showed significant differences in species richness related to the distance from the road across elevation (adjusted *R*² = 0.38, *p* = .034), where native species richness was lower in the *roadside* plots compared to *near* and *far* plots (Table 3). For non‐native species, the elevational trends were always quadratic (*roadside*:* R*² = 0.55, *p* < .001; *near*:* R*² = 0.39, *p* = .010; *far*:* R*² = 0.33, *p* = .025) (Figure 3b, Table 2). Student's *t* test for fit parameters to quadratic model indicated significant differences on species richness–elevation relationship among the three group of plots (*roadside*,* near,* and *far*) (Table 3), with non‐native species decreasing in number with increasing distance from the road across elevations.

**Table 3:** ANCOVA results for native species richness in relation to distance from the road and elevation. Parameter data, standard error, and significance for species richness in subtransects of *roadside*,* near,* and *far* and *t* test results between groups, considering the parameters of each equation and their standard errors

Source	Type III sum of squares	*df*	Mean square	*F*	Sig.
ANCOVA					
location a elevation	872.9	2	436.5	3.6	0.03
location	1,266.9	2	633.4	5.2	0.01
elevation	2,723.6	1	2,723.6	22.5	0
Error	6,540.8	54	121.1		
Total	153,430	60			
Total corrected	11,517.9	59			

Parameter	Groups	Statistic value *t* test	Sig. (*p* < .01)
*t* test			
For the coefficients of quadratic terms			
a	Roadside‐near	−1,829.1^a^	*p* < .01
	Roadside‐far	−2,635.4^a^	*p* < .01
	Near‐far	−806.4^a^	*p* < .01
For the coefficients of first‐degree terms			
b	Roadside‐near	354.8^a^	*p* < .01
	Roadside‐far	512.8^a^	*p* < .01
	Near‐far	182.7^a^	*p* < .01

a significant.

**Figure 4:**
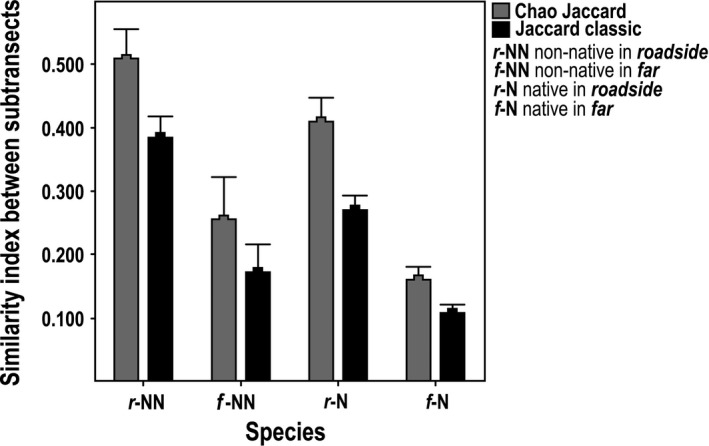
Similarity analysis of Chao‐Jaccard and classic Jaccard indices, between assemblages of species, native and non‐native, in subtransects roadside and far of road, along the elevational gradient

Similarity analysis with Chao‐Jaccard and Jaccard indices showed the same consistent results, the variation in floristic composition between roadside and far from the road communities across the elevational gradient. The roadside plots always showed greater similarity in the composition of native and non‐native species than in the plots far from road, where the similarity was lower (Figure 4).

### 3.4 Origin of non‐native species in the gradient

**Figure 5:**
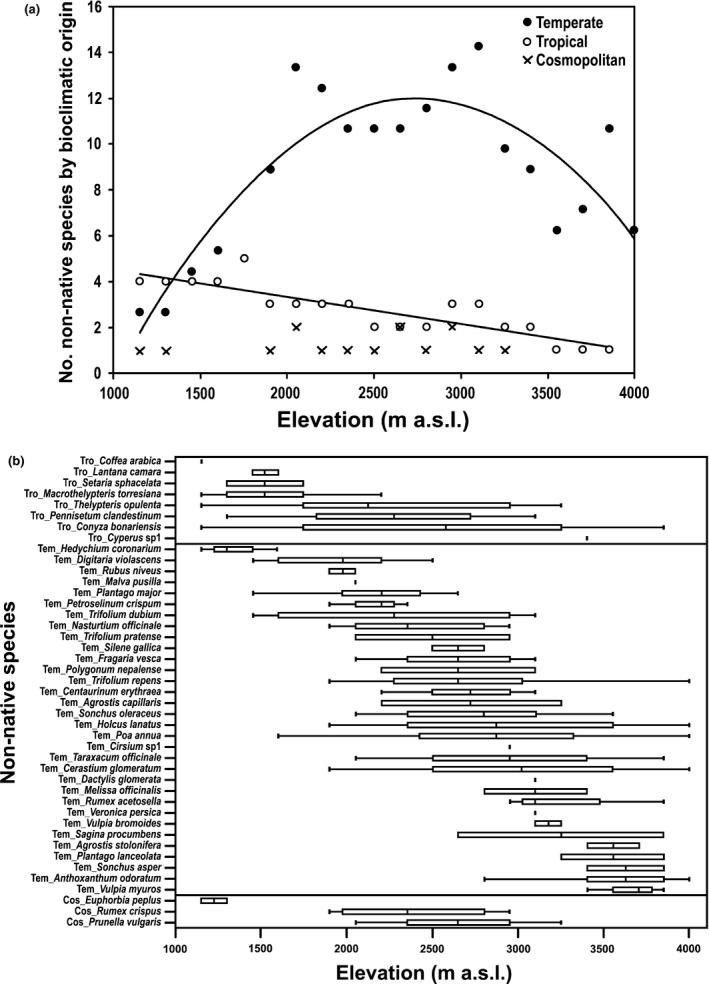
(a) Distribution of non‐native species respect their bioclimatic origin, located in the elevational gradient (regression results in Table 2). (b) Elevational distribution range of 43 non‐native species located in the 20 transects, with their maximum, minimum, and median

Thirty‐two non‐native species (74%) came from temperate regions, whereas eight species (19%) were from tropical regions and only three species (7%) had a cosmopolitan distribution. The results of the regression models for these species showed a quadratic trend for the group from temperate regions, with an increase in the number of species at middle elevations. In contrast, species of tropical origin showed linear monotonic decrease with elevations, whereas the cosmopolitan showed no trends (Figure 5a, Table 2).

Non‐native species of temperate origin showed wider elevational ranges than tropical ones, most located between 2,050 and 3,250 m a.s.l.; *Poa annua* had the broadest elevational range 1,600–4,000 m a.s.l., *Cerastium glomeratum* and *Holcus lanatus* were present between 1,900‐4,000 m a.s.l. *Taraxacum officinale* and 2,050–3,850 m a.s.l. Species of tropical origin were most common at lower elevations (1,150–1,750 m a.s.l.), where *Conyza bonariensis* and *Thelypteris opulenta* had the broader elevational ranges: 1,150–3,400 m a.s.l. and 1,150–3,250 m a.s.l., respectively. The cosmopolitan species were present from low elevations, as the species *Euphorbia peplus* (1,150 m a.s.l.), to high elevations such as *Prunella vulgaris* (3,250 m a.s.l.) (Figure 5b).

## 4 DISCUSSION

We found that along a wide elevational gradient located in a tropical continental area of South America, native and non‐native plant species showed different distributional patterns that also differ from what has been reported for temperate areas. Disturbance, assessed as the distance to road, and the temperate or tropical origin of non‐native species also play important roles in determining the species richness and the shape of the elevational distribution of non‐native species.

### 4.1 Elevational distribution patterns

The diversity of native species found in this study is remarkably high (728 species), considering that the total number of species recorded for the entire Ecuadorian Amazon is 4,857 (Jørgensen & León‐Yánez, 1999), and that we sampled only the mid and upper vegetation belts. The number of non‐native species found here (43 species, 5.9% of the total) represents a relatively low value when compared to other studies conducted in the tropical and subtropical mountainous islands such as Tenerife (38 non‐native species along a gradient of 2,200‐m elevation) and Hawaii (162 non‐native species along a gradient of 4,000‐m elevation). Given the high number of native plant species, the proportion of non‐native species is significantly lower than those reported for mountains in temperate regions (Alexander et al., 2011; Seipel et al., 2012). Thus, high‐elevation habitats of the tropical Ecuadorian Andes as a general rule appear to be less invaded than other mountains; although in research over the Chimborazo, between 3,800‐ and 5,200‐m elevation, it was determined that the vegetation assessed by Humboldt, between the years 1769 and 1859, is altered currently because the intensive land use change added to climatic change that have led to the change of diversity and species distribution range in high‐elevation habitats of this mountain (Morueta‐Holme et al., 2015).

Some studies show that in tropical regions, the elevational distribution of species, seemed to follows a hump‐shaped curve (Balslev 1988, Arévalo et al., 2005; Pauchard et al., 2009; Kessler et al., 2011). For the Eastern Cordillera of Ecuador, the highest number of species has been found at middle elevation (between 1,000–1,500 m a.s.l.), declines from 1,000 m a.s.l. to lowlands and from 1,500 m a.s.l. to higher elevations (Gentry, 1995; Jørgensen & León‐Yánez, 1999). Balslev (1988) when conducting a regional assessment of plant species richness in Ecuador found that the largest number of species is found between 900 and 3,000 m a.s.l. However, in the present study, we found a linear monotonic decrease of native species, and this pattern was generated due to the elevational extent that comprised our gradient. The lowest elevation we studied was 1,150 m a.s.l., located mostly in the Humid Temperate Region, where is the highest diversity of Ecuador, with intermediate temperatures and high humidity (Cañadas, 1983). Nogués‐Bravo et al. (2008) reported that the form of the elevational gradient of species richness depends not only on the scale, but also on the length of the gradient (see also Kessler et al., 2011). Thus, the inclusion of sites from lower elevations might produce that expected hump‐shaped pattern for native species. Notwithstanding, the fact that native species richness showed a monotonic decline with elevation points to the elevational increase in climate harshness as an important filter as it has been widely discussed in other studies (Grytnes & Vetaas, 2002; Grytnes et al., 2006; Kluge et al., 2006; Nogués‐Bravo et al. 2008, Kessler et al., 2011).

The unimodal (hump‐shaped) distributional pattern found for non‐native species suggest that the best environmental conditions for their successful establishment are located in middle elevations. On the one hand, the decrease in the number of non‐natives toward higher elevations can be easily understood by the climate filter as occurs for natives (see also Alexander et al., 2011); further higher elevations in our study sites corresponded to a protected area with a very sparse human presence, and as determined by Traux, Vankat, and Schaefer (1992) and Pauchard et al. (2009), elevation is associated with a gradient of land use, which is also an important determinant of the distribution of non‐native species. On the other hand, the areas with high anthropogenic disturbance due to the presence of human settlements in Ecuador are commonly found around 2,900 m a.s.l.

The decrease in number of non‐native toward lower elevation could be related to the biotic resistance theory proposed by Elton (1958), who determined that the native species‐rich communities have more resistance to invasions of exotic species (Rejmánek, 1989). At low elevations, communities contain more native species, having higher competitive ability and lacking available empty niches for the recruitment of new species; therefore, the number of non‐native species that can be established at these low elevation sites will be low (Herben, Mandák, Bímová, & Munzbergová, 2004). Further, the hump‐shaped pattern could also be related to the fact that most of the non‐native species come from temperate regions, finding their optimum of distribution at medium elevations.

### 4.2 The effect of disturbance: distance from the road

Throughout the elevational gradient, we found that the roadsides were highly disturbed habitats that favored the establishment of non‐native species and the decline of native diversity. This is mainly because in the roadside, there are more disturbances and removal of vegetation due to maintenance of the road and the traffic, with a concomitant increase propagule pressure, generate advantages for invasion of non‐natives (Seipel et al., 2012). While increasing distance from the road disturbances decreased promoting communities with fewer non‐native species and more native species across elevation. The *roadside* plots have fewer species turnover between them; therefore, they have a more similar floristic composition, with non‐native species that are often present across the entire altitudinal gradient. Among *far* plots, the floristic similarity is lower, because the identity of the species varies with a greater turnover along the gradient. The *roadside* habitats are more homogeneous in structure because species adapted to disturbed environments are sustained within its composition. It is well known that roads are promoters for the entry of non‐native species (Arteaga et al., 2009; Becker et al., 2005; Otto et al., 2014) and act as biological corridors and dispersal agents of their propagules, providing suitable habitat for their establishment (Pauchard & Alaback, 2004), while in distant habitats the species composition responds to local heterogeneity patterns by low intensity of disturbance.

### 4.3 Origin of non‐native species in the gradient

Similar to McDougall et al. (2011), in this study most of the non‐native species richness belong to Poaceae and Asteraceae families. Probably this is related to fact that these families contain annuals or perennials herbaceous species with broad range of climate distribution. Our results on the distribution patterns of non‐native species along the elevational gradient in relation to their biogeographical origin concurs with other studies (Arévalo et al., 2005; Arteaga et al., 2009; Becker et al., 2005; Daehler, 2005; Pauchard & Alaback, Pauchard et al., 2009). The distribution of non‐native species across an elevational gradient is determined by the similarity of climatic conditions between the native range of a non‐native species and the area of introduction (Seipel et al., 2012). Accordingly, we found that species from tropical and subtropical areas were located in the lower elevations, while species of temperate origin showed their optimum at medium and high elevations (Becker et al., 2005; Pauchard & Alaback, 2004; Pauchard et al., 2009). When the elevation increases, the species of tropical origin are replaced by species of temperate affinities (Wester & Juvik, 1983). The species of tropical and subtropical origin have narrow thermal tolerance and are limited to lower elevations because of low temperatures and low tolerance to frost and environmental stress in high elevations (Arteaga et al., 2009; Becker et al., 2005; Daehler, 2005; Pauchard & Alaback, 2004). Along the elevational gradient studied, there is a predominance of temperate origin species, with large overlapping ranges of distribution, suggesting that in general these species can tolerate wide climate variation in new areas of introduction (Seipel et al., 2012). This might be observed in species of temperate origin such as *Cerastium glomeratum*,* Poa annua*,* Holcus lanatus, Trifolium repens*,* Plantago lanceolata,* and *Rumex acetosella* which have a wide distribution along the gradient, and had been frequently found in similar other studies in tropical or temperate mountains (McDougall et al., 2011; Morueta‐Holme et al., 2015). Interestingly, the higher part of the tropical elevational gradient follows the uni‐directional ecological filtering described mostly for temperate regions (Alexander et al., 2011). In addition, the dominance of species from temperate origin could be due to intentional and accidental introductions, mostly after the European colonization (McDougall et al., 2011). Cosmopolitan species were few (3 species) and preferably located in the middle elevational zones, suggesting that there is low propagule pressure for this species in our studied region. According to Alexander et al. (2011), the climatic niche of some non‐native species may have changed in the introduced range, as seen in species that have expanded their range. In our case, *Poa annua* that comes from a temperate climate was found from 1,600 to 4,000 m a.s.l., and *Conyza bonariensis* of tropical origin was found among 1,150 to 3,850 m a.s.l., indicating the ability to colonize zones with different climate conditions than those occurring in their native ranges.

## 5 CONCLUSIONS

Although the diversity of native species was higher than that of non‐native species along the entire tropical elevational gradient studied, non‐native species were present in all sites, especially along roadside habitats. This type of disturbance determined plant species distribution across elevations, generating a unique plant assemblage in the roadsides with a higher proportion of non‐native plants than less disturbed areas.

While native species have a monotonic linear decline of richness with increasing elevation, non‐native species showed a hump‐shaped pattern, with increases in richness in middle elevations. These contrasting patterns suggest that factors involved in shaping the distribution of native plants differ from that of the non‐natives. This is particularly noticeable at lower elevations where either lower density of human settlements and disturbances, or higher resistance of a highly diverse native flora decrease the presence of non‐native species. In the higher end of the gradient, patterns of non‐native and native species distributions tend to equate to those found in temperate regions of the world. Both the presence of disturbance and the biogeographic origin of the non‐native species have important influence in their elevational distribution, with European species becoming more relevant at mid elevations and reducing their abundance in the highest areas. While disturbance is a promoter of the success of non‐native species, the biogeographic origins appear to play a larger role in determining the elevational range that non‐native species can occupy in the mountains of Ecuador.

Our study highlights the importance of having more data on how native and non‐native species are distributed in tropical regions of the world. Although there are some commonalities with temperate regions, which have been profusely studied, there are striking differences in how species are distributed in the lower, warmer zones of these mountains. Understanding the interaction between non‐native and native plants and the role of elevation and disturbance in shaping their distribution will be critical to better conserve biodiversity in tropical areas under an increasing threat by human development.
