# Supplementary material for: Natives and non‐natives plants show different responses to elevation and disturbance on the tropical high Andes of Ecuador
Source: Ecol Evol. 2017 Aug 30;7(19):7909–19. doi: 10.1002/ece3.3270 (PMC5632626; doi:10.1002/ece3.3270)
Supplement: Supplementary file 1 [file ECE3-7-7909-s001.doc]

**Appendix 1. Native and non-native species found along the mountain road at 20 sampled elevations points.**

| **Specie** | | **Habit** | **Status** | **Biogeographic origin** | **Phytogeographic region** | | **Latitudinal area** | **Number of individuals** |
| --- | --- | --- | --- | --- | --- | --- | --- | --- |
| *Acaena elongata* | | herb | NAT | NA | NA | |  | 18 |
| *Acaena ovalifolia* | | herb | NAT | NA | NA | |  | 29 |
| *Acalypha benensis* | | treelet | NAT | NA | NA | |  | 1 |
| *Acalypha cuneata* | | treelet | NAT | NA | NA | |  | 15 |
| *Acalypha diversifolia* | | shrub | NAT | NA | NA | |  | 7 |
| *Acalypha macrostachya* | | shrub | NAT | NA | NA | |  | 1 |
| *Acalypha padifolia* | | shrub | NAT | NA | NA | |  | 4 |
| *Acalypha platyphylla* | | shrub | NAT | NA | NA | |  | 1 |
| *Achyrocline alata* | | herb | NAT | NA | NA | |  | 53 |
| *Achyrocline satureioides* | | herb | NAT | NA | NA | |  | 12 |
| *Aciotis rubricaulis* | | herb | NAT | NA | NA | |  | 26 |
| *Acroceras zizanioides* | | herb | NAT | NA | NA | |  | 16 |
| *Adiantum concinnum* | | ferm | NAT | NA | NA | |  | 25 |
| *Aegopogon cenchroides* | | herb | NAT | NA | NA | |  | 16 |
| *Aetanthus nodosus* | | shrub | NAT | NA | NA | |  | 1 |
| *Aetheolaena mojandensis* | | shrub | E | NA | NA | |  | 2 |
| *Ageratina pichinchensis* | | herb | NAT | NA | NA | |  | 108 |
| *Ageratina rhypodes* | | herb | E | NA | NA | |  | 27 |
| *Ageratina sodiroi* | | herb | E | NA | NA | |  | 119 |
| *Agouticarpa isernii* | | shrub | NAT | NA | NA | |  | 1 |
| *Agrostis breviculmis* | | herb | NAT | NA | NA | |  | 26 |
| *Agrostis capillaris* | | herb | IN | Eurasia | Paleartic | | Temperate | 9 |
| *Agrostis perennans* | | herb | NAT | NA | NA | |  | 13 |
| *Agrostis* cf. *stolonifera* | | herb | IN | Europe, Asia | Paleartic | | Temperate | 5 |
| *Agrostis tolucensis* | | herb | NAT | NA | NA | |  | 54 |
| *Alchornea latifolia* | | tree | NAT | NA | NA | |  | 10 |
| *Allophylus floribundus* | | tree | NAT | NA | NA | |  | 5 |
| *Allophylus stenodictyus* | | tree | NAT | NA | NA | |  | 1 |
| *Alloplectus weirii* | | herb | NAT | NA | NA | |  | 22 |
| *Alnus acuminata* | | tree | NAT | NA | NA | |  | 34 |
| *Alonsoa meridionalis* | | herb | NAT | NA | NA | |  | 13 |
| *Alsophila erinacea* | | herb | NAT | NA | NA | |  | 11 |
| *Alternanthera mexicana* | | herb | NAT | NA | NA | |  | 3 |
| *Alternanthera porrigens* | | herb | NAT | NA | NA | |  | 6 |
| *Amphilophium aschersonii* | | liana | NAT | NA | NA | |  | 6 |
| *Amphilophium crucigerum* | | liana | NAT | NA | NA | |  | 1 |
| *Andropogon bicornis* | | herb | NAT | NA | NA | |  | 9 |
| *Aniba hostmanniana* | | tree | NAT | NA | NA | |  | 1 |
| *Anthoxanthum odoratum* | | herb | IN | Eurasia | Paleartic | | Temperate | 50 |
| *Anthurium andreanum* | | herb | NAT | NA | NA | |  | 16 |
| *Anthurium flavolineatum* | | herb | NAT | NA | NA | |  | 3 |
| *Anthurium* cf. *formosum* | | herb | NAT | NA | NA | |  | 67 |
| *Aphelandra acanthus* | | shrub | NAT | NA | NA | |  | 6 |
| *Aphelandra rosulata* | | herb | NAT | NA | NA | |  | 3 |
| *Arenaria lanuginosa* | | herb | NAT | NA | NA | |  | 33 |
| *Arenaria parvifolia* | | herb | NAT | NA | NA | |  | 37 |
| *Asplenium auritum* | | ferm | NAT | NA | NA | |  | 7 |
| *Asplenium flabellatum* | | ferm | NAT | NA | NA | |  | 7 |
| *Asplenium haenkeanum* | | ferm | NAT | NA | NA | |  | 3 |
| *Asplenium monanthes* | | ferm | NAT | NA | NA | |  | 1 |
| *Astrocaryum chambira* | | tree | NAT | NA | NA | |  | 1 |
| *Axonopus compressus* | | herb | NAT | NA | NA | |  | 19 |
| *Axonopus micay* | | herb | NAT | NA | NA | |  | 60 |
| *Axonopus scoparius* | | herb | NAT | NA | NA | |  | 82 |
| *Azorella aretioides* | | herb | NAT | NA | NA | |  | 1 |
| *Azorella pedunculata* | | herb | NAT | NA | NA | |  | 30 |
| *Baccharis arbutifolia* | | shrub | NAT | NA | NA | |  | 3 |
| *Baccharis buxifolia* | | shrub | NAT | NA | NA | |  | 13 |
| *Baccharis genistelloides* | | herb | NAT | NA | NA | |  | 10 |
| *Baccharis latifolia* | | shrub | NAT | NA | NA | |  | 233 |
| *Baccharis* cf. *nitida* | | shrub | NAT | NA | NA | |  | 6 |
| *Baccharis prunifolia* | | shrub | NAT | NA | NA | |  | 9 |
| *Baccharis trinervis* | | shrub | NAT | NA | NA | |  | 6 |
| *Banara guianensis* | | shrub | NAT | NA | NA | |  | 22 |
| *Barnadesia arborea* | | shrub | NAT | NA | NA | |  | 8 |
| *Bartsia inaequalis* | | herb | NAT | NA | NA | |  | 4 |
| *Bartsia stricta* | | herb | NAT | NA | NA | |  | 6 |
| *Begonia acerifolia* | | herb | E | NA | NA | |  | 15 |
| *Begonia dodsonii* | | herb | E | NA | NA | |  | 11 |
| *Begonia glabra* | | herb | NAT | NA | NA | |  | 6 |
| *Begonia maurandiae* | | herb | NAT | NA | NA | |  | 7 |
| *Begonia parviflora* | | treelet | NAT | NA | NA | |  | 21 |
| *Bellucia pentamera* | | tree | NAT | NA | NA | |  | 1 |
| *Besleria aggregata* | | shrub | NAT | NA | NA | |  | 10 |
| *Besleria angustiflora* | | shrub | NAT | NA | NA | |  | 1 |
| *Besleria barclayi* | | shrub | NAT | NA | NA | |  | 3 |
| *Besleria comosa* | | shrub | E | NA | NA | |  | 5 |
| *Besleria quadrangulata* | | shrub | E | NA | NA | |  | 1 |
| *Besleria stricta* | | shrub | NAT | NA | NA | |  | 1 |
| *Bidens andicola* | | herb | NAT | NA | NA | |  | 29 |
| *Bidens pilosa* | | herb | NAT | NA | NA | |  | 61 |
| *Blakea subvaginata* | | treelet | E | NA | NA | |  | 2 |
| *Blechnum chilense* | | ferm | NAT | NA | NA | |  | 12 |
| *Blechnum cordatum* | | ferm | NAT | NA | NA | |  | 45 |
| *Blechnum* cf. *divergens* | | ferm | NAT | NA | NA | |  | 28 |
| *Blechnum lechleri* | | ferm | NAT | NA | NA | |  | 4 |
| *Blechnum loxense* | | ferm | NAT | NA | NA | |  | 12 |
| *Blechnum occidentale* | | ferm | NAT | NA | NA | |  | 3 |
| *Blechnum stipitellatum* | | ferm | NAT | NA | NA | |  | 4 |
| *Bocconia integrifolia* | | treelet | NAT | NA | NA | |  | 1 |
| *Boehmeria caudata* | | shrub | NAT | NA | NA | |  | 1 |
| *Boehmeria ramiflora* | | shrub | NAT | NA | NA | |  | 34 |
| *Bomarea multiflora* | | liana | NAT | NA | NA | |  | 21 |
| *Borreria ocymoides* | | herb | NAT | NA | NA | |  | 50 |
| *Borreria remota* | | herb | NAT | NA | NA | |  | 21 |
| *Botrychium schaffneri* | | ferm | NAT | NA | NA | |  | 23 |
| *Botrychium virginianum* | | ferm | NAT | NA | NA | |  | 4 |
| *Briza monandra* | | herb | NAT | NA | NA | |  | 2 |
| *Bromus lanatus* | | herb | NAT | NA | NA | |  | 57 |
| *Brugmansia* cf. *sanguinea* | | treelet | NAT | NA | NA | |  | 3 |
| *Brunellia acostae* | | tree | NAT | NA | NA | |  | 11 |
| *Bunchosia argentea* | | tree | NAT | NA | NA | |  | 1 |
| *Burmeistera crispiloba* | | shrub | E | NA | NA | |  | 1 |
| *Burmeistera* cf. *glabrata* | | shrub | NAT | NA | NA | |  | 2 |
| *Burmeistera multiflora* | | shrub | NAT | NA | NA | |  | 4 |
| *Burmeistera refracta* | | shrub | E | NA | NA | |  | 16 |
| *Caladium bicolor* | | herb | NAT | NA | NA | |  | 8 |
| *Calamagrostis fibrovaginata* | | herb | NAT | NA | NA | |  | 5 |
| *Calamagrostis intermedia* | | herb | NAT | NA | NA | |  | 7 |
| *Calamagrostis planifolia* | | herb | NAT | NA | NA | |  | 62 |
| *Calatola costaricensis* | | tree | NAT | NA | NA | |  | 1 |
| *Calceolaria chelidonioides* | | herb | NAT | NA | NA | |  | 3 |
| *Calceolaria crenata* | | herb | NAT | NA | NA | |  | 19 |
| *Calceolaria lamiifolia* | | herb | NAT | NA | NA | |  | 1 |
| *Calceolaria mexicana* | | herb | NAT | NA | NA | |  | 17 |
| *Calceolaria perfoliata* | | herb | NAT | NA | NA | |  | 34 |
| *Calceolaria tripartita* | | herb | NAT | NA | NA | |  | 3 |
| *Calliandra trinervia* | | tree | NAT | NA | NA | |  | 2 |
| *Campyloneurum amphostenon* | | ferm | NAT | NA | NA | |  | 26 |
| *Campyloneurum angustifolium* | | ferm | NAT | NA | NA | |  | 6 |
| *Campyloneurum solutum* | | ferm | NAT | NA | NA | |  | 8 |
| *Capparis detonsa* | | tree | NAT | NA | NA | |  | 1 |
| *Carex bonplandii* | | herb | NAT | NA | NA | |  | 32 |
| *Carex brachycalama* | | herb | NAT | NA | NA | |  | 1 |
| *Carex lemanniana* | | herb | NAT | NA | NA | |  | 29 |
| *Carex pichinchensis* | | herb | NAT | NA | NA | |  | 22 |
| *Carica × heilbornii nm chrysopetala* | | treelet | NAT | NA | NA | |  | 1 |
| *Carludovica palmata* | | herb | NAT | NA | NA | |  | 3 |
| *Casearia aculeata* | | shrub | NAT | NA | NA | |  | 2 |
| *Castilleja arvensis* | | herb | NAT | NA | NA | |  | 18 |
| *Castilleja fissifolia* | | herb | NAT | NA | NA | |  | 20 |
| *Cavendishia bracteata* | | shrub | NAT | NA | NA | |  | 4 |
| *Cayaponia duckei* | | liana | NAT | NA | NA | |  | 2 |
| *Cecropia* cf. *angustifolia* | | tree | NAT | NA | NA | |  | 1 |
| *Cecropia ficifolia* | | tree | NAT | NA | NA | |  | 6 |
| *Cecropia membranacea* | | tree | NAT | NA | NA | |  | 2 |
| *Cedrela odorata* | | tree | NAT | NA | NA | |  | 3 |
| *Centaurium erythraea* | | herb | IN | Mediterranean | Paleartic | | Temperate | 40 |
| *Centropogon caoutchouc* | | shrub | NAT | NA | NA | |  | 11 |
| *Centropogon cazaletii* | | shrub | E | NA | NA | |  | 2 |
| *Centropogon curvatus* | | shrub | NAT | NA | NA | |  | 1 |
| *Centropogon granulosus* | | shrub | NAT | NA | NA | |  | 1 |
| *Centropogon* cf. *medusa* | | shrub | E | NA | NA | |  | 4 |
| *Centropogon preslii* | | shrub | NAT | NA | NA | |  | 14 |
| *Cerastium candicans* | | herb | NAT | NA | NA | |  | 1 |
| *Cerastium glomeratum* | | herb | IN | Eurasia | Paleartic | | Temperate | 87 |
| *Ceratochloa pitensis* | | herb | NAT | NA | NA | |  | 19 |
| *Cestrum peruvianum* | | shrub | NAT | NA | NA | |  | 1 |
| *Cestrum tomentosum* | | shrub | NAT | NA | NA | |  | 20 |
| *Chrysochlamys* cf. *dependens* | | treelet | NAT | NA | NA | |  | 1 |
| *Chrysochlamys membranacea* | | treelet | NAT | NA | NA | |  | 1 |
| *Chusquea scandens* | | herb | NAT | NA | NA | |  | 76 |
| *Chusquea uniflora* | | herb | NAT | NA | NA | |  | 4 |
| *Cirsium* sp1. | | herb | IN | NA | NA | | Temperate | 1 |
| *Cissus erosa* | | liana | NAT | NA | NA | |  | 7 |
| *Clematis haenkeana* | | liana | NAT | NA | NA | |  | 6 |
| *Cleome anomala* | | shrub | NAT | NA | NA | |  | 12 |
| *Cleome glandulosa* | | shrub | NAT | NA | NA | |  | 1 |
| *Clethra fagifolia* | | tree | NAT | NA | NA | |  | 2 |
| *Clibadium grandifolium* | | shrub | NAT | NA | NA | |  | 2 |
| *Clibadium microcephalum* | | shrub | E | NA | NA | |  | 17 |
| *Clibadium peruvianum* | | shrub | NAT | NA | NA | |  | 3 |
| *Clibadium sprucei* | | shrub | E | NA | NA | |  | 9 |
| *Clidemia dentata* | | shrub | NAT | NA | NA | |  | 27 |
| *Clidemia pilosa* | | shrub | NAT | NA | NA | |  | 5 |
| *Clidemia sprucei* | | shrub | NAT | NA | NA | |  | 2 |
| *Clinopodium nubigenum* | | herb | NAT | NA | NA | |  | 16 |
| *Clusia decussata* | | shrub | NAT | NA | NA | |  | 1 |
| *Clusia laxiflora* | | shrub | NAT | NA | NA | |  | 6 |
| *Coccoloba* cf. *coronata* | | tree | NAT | NA | NA | |  | 1 |
| *Coffea arabica* | | shrub | IN | Africa | Paleotropical | | Tropical | 2 |
| *Colobanthus quitensis* | | herb | NAT | NA | NA | |  | 7 |
| *Columnea albiflora* | | herb | E | NA | NA | |  | 11 |
| *Columnea ericae* | | herb | NAT | NA | NA | |  | 9 |
| *Columnea guttata* | | herb | NAT | NA | NA | |  | 10 |
| *Columnea isernii* | | herb | NAT | NA | NA | |  | 1 |
| *Columnea tandapiana* | | herb | NAT | NA | NA | |  | 33 |
| *Commelina obliqua* | | herb | NAT | NA | NA | |  | 3 |
| *Commelina quitensis* | | herb | NAT | NA | NA | |  | 54 |
| *Conyza bonariensis* | | herb | IN | Central America | Neotropical | | Tropical | 170 |
| *Conyza uliginosa* | | herb | NAT | NA | NA | |  | 15 |
| *Cordia alliodora* | | tree | NAT | NA | NA | |  | 1 |
| *Coriaria ruscifolia* | | shrub | NAT | NA | NA | |  | 67 |
| *Cortaderia nitida* | | herb | NAT | NA | NA | |  | 53 |
| *Cortaderia sericantha* | | herb | NAT | NA | NA | |  | 31 |
| *Costus* cf. *longibracteolatus* | | herb | NAT | NA | NA | |  | 5 |
| *Costus scaber* | | herb | NAT | NA | NA | |  | 11 |
| *Cotula mexicana* | | herb | NAT | NA | NA | |  | 90 |
| *Crotalaria nitens* | | shrub | NAT | NA | NA | |  | 6 |
| *Croton lechleri* | | tree | NAT | NA | NA | |  | 5 |
| *Cupania latifolia* | | treelet | NAT | NA | NA | |  | 1 |
| *Cuphea* cf. *bombonasae* | | shrub | NAT | NA | NA | |  | 1 |
| *Cuphea racemosa* | | herb | NAT | NA | NA | |  | 31 |
| *Cuphea strigulosa* | | herb | NAT | NA | NA | |  | 6 |
| *Cyathea caracasana* | | treelet | NAT | NA | NA | |  | 1 |
| *Cyathea tortuosa* | | treelet | NAT | NA | NA | |  | 3 |
| *Cybianthus* cf. *timanae* | | shrub | NAT | NA | NA | |  | 3 |
| *Cyclanthus bipartitus* | | herb | NAT | NA | NA | |  | 10 |
| *Cynanchum formosum* | | liana | NAT | NA | NA | |  | 1 |
| *Cyperus aggregatus* | | herb | NAT | NA | NA | |  | 16 |
| *Cyperus chalaranthus* | | herb | NAT | NA | NA | |  | 3 |
| *Cyperus luzulae* | | herb | NAT | NA | NA | |  | 15 |
| *Cyperus simplex* | | herb | NAT | NA | NA | |  | 1 |
| *Cyperus* sp1. | | herb | IN | NA | NA | | Tropical | 4 |
| *Cystopteris fragilis* | | ferm | NAT | NA | NA | |  | 38 |
| *Dactylis glomerata* | | herb | IN | Eurasia | Paleartic | | Temperate | 14 |
| *Danaea moritziana* | | ferm | NAT | NA | NA | |  | 12 |
| *Danaea* cf. *nodosa* | | ferm | NAT | NA | NA | |  | 11 |
| *Daucus montanus* | | herb | NAT | NA | NA | |  | 10 |
| *Dendropanax caucanus* | | treelet | NAT | NA | NA | |  | 8 |
| *Dendrophorbium* cf. *balsapampae* | | treelet | E | NA | NA | |  | 4 |
| *Dendrophorbium tipocochensis* | | treelet | E | NA | NA | |  | 3 |
| *Dennstaedtia* cf. *auriculata* | | ferm | NAT | NA | NA | |  | 1 |
| *Dennstaedtia cornuta* | | ferm | NAT | NA | NA | |  | 3 |
| *Dennstaedtia dissecta* | | ferm | NAT | NA | NA | |  | 7 |
| *Desmodium adscendens* | | herb | NAT | NA | NA | |  | 32 |
| *Puya hamata* | | herb | NAT | NA | NA | |  | 2 |
| *Desmodium campyloclados* | | liana | NAT | NA | NA | |  | 8 |
| *Desmodium purpusii* | | liana | NAT | NA | NA | |  | 12 |
| *Dieffenbachia harlingii* | | herb | NAT | NA | NA | |  | 8 |
| *Digitaria ciliaris* | | herb | NAT | NA | NA | |  | 12 |
| *Digitaria violascens* | | herb | IN | Asia | Paleartic | | Temperate | 16 |
| *Dioscorea sprucei* | | liana | NAT | NA | NA | |  | 7 |
| *Diplazium alienum* | | ferm | NAT | NA | NA | |  | 10 |
| *Diplazium costale* | | ferm | NAT | NA | NA | |  | 17 |
| *Diplazium expansum* | | ferm | NAT | NA | NA | |  | 7 |
| *Diplazium grandifolium* | | ferm | NAT | NA | NA | |  | 2 |
| *Diplostephium ericoides* | | shrub | E | NA | NA | |  | 4 |
| *Diplostephium glandulosum* | | shrub | NAT | NA | NA | |  | 2 |
| *Diplostephium rupestre* | | shrub | NAT | NA | NA | |  | 21 |
| *Diplostephium schultzii* | | shrub | NAT | NA | NA | |  | 5 |
| *Disterigma acuminatum* | | shrub | NAT | NA | NA | |  | 5 |
| *Disterigma alaternoides* | | shrub | NAT | NA | NA | |  | 1 |
| *Disterigma empetrifolium* | | shrub | NAT | NA | NA | |  | 43 |
| *Dolichandra uncata* | | liana | NAT | NA | NA | |  | 7 |
| *Dorobaea pimpinellifolia* | | herb | NAT | NA | NA | |  | 8 |
| *Drymaria* cf. *cordata* | | herb | NAT | NA | NA | |  | 44 |
| *Drymaria ovata* | | herb | NAT | NA | NA | |  | 24 |
| *Drymonia serrulata* | | herb | NAT | NA | NA | |  | 3 |
| *Drymonia tenuis* | | herb | NAT | NA | NA | |  | 2 |
| *Drymonia turrialvae* | | herb | NAT | NA | NA | |  | 10 |
| *Drymonia variegata* | | herb | NAT | NA | NA | |  | 3 |
| *Drymonia warscewicziana* | | herb | NAT | NA | NA | |  | 6 |
| *Dussia tessmannii* | | tree | NAT | NA | NA | |  | 1 |
| *Dysopsis paucidentata* | | herb | NAT | NA | NA | |  | 1 |
| *Elaeagia mariae* | | tree | NAT | NA | NA | |  | 2 |
| *Elaphoglossum albescens* | | ferm | NAT | NA | NA | |  | 6 |
| *Elaphoglossum ambiguum* | | ferm | NAT | NA | NA | |  | 2 |
| *Elaphoglossum castaneum* | | ferm | NAT | NA | NA | |  | 2 |
| *Elaphoglossum cuspidatum* | | ferm | NAT | NA | NA | |  | 2 |
| *Elaphoglossum engelii* | | ferm | NAT | NA | NA | |  | 4 |
| *Elaphoglossum erinaceum* | | ferm | NAT | NA | NA | |  | 1 |
| *Elaphoglossum gayanum* | | ferm | NAT | NA | NA | |  | 24 |
| *Elaphoglossum glabellum* | | ferm | NAT | NA | NA | |  | 6 |
| *Elaphoglossum lindigii* | | ferm | NAT | NA | NA | |  | 6 |
| *Elaphoglossum minutum* | | ferm | NAT | NA | NA | |  | 5 |
| *Elaphoglossum ovatum* | | ferm | NAT | NA | NA | |  | 4 |
| *Elaphoglossum vulcanicum* | | ferm | NAT | NA | NA | |  | 11 |
| *Elaphoglossum yatesii* | | ferm | NAT | NA | NA | |  | 5 |
| *Eleocharis acicularis* | | herb | NAT | NA | NA | |  | 18 |
| *Eleocharis bonariensis* | | herb | NAT | NA | NA | |  | 13 |
| *Eleocharis dombeyana* | | herb | NAT | NA | NA | |  | 9 |
| *Elymus cordilleranus* | | herb | NAT | NA | NA | |  | 13 |
| *Epidendrum fimbriatum* | | herb | NAT | NA | NA | |  | 10 |
| *Epilobium denticulatum* | | herb | NAT | NA | NA | |  | 43 |
| *Equisetum bogotense* | | herb | NAT | NA | NA | |  | 114 |
| *Equisetum giganteum* | | herb | NAT | NA | NA | |  | 6 |
| *Erato polymnioides* | | shrub | NAT | NA | NA | |  | 74 |
| *Erechtites valerianifolius* | | herb | NAT | NA | NA | |  | 2 |
| *Erigeron ecuadoriensis* | | herb | NAT | NA | NA | |  | 43 |
| *Eryngium humile* | | herb | NAT | NA | NA | |  | 1 |
| *Erythrina edulis* | | tree | NAT | NA | NA | |  | 19 |
| *Escallonia myrtilloides* | | shrub | NAT | NA | NA | |  | 26 |
| *Eschweilera caudiculata* | | treelet | NAT | NA | NA | |  | 2 |
| *Esenbeckia amazonica* | | tree | NAT | NA | NA | |  | 1 |
| *Eugenia dibrachiata* | | treelet | NAT | NA | NA | |  | 2 |
| *Eugenia florida* | | treelet | NAT | NA | NA | |  | 3 |
| *Euphorbia laurifolia* | | treelet | NAT | NA | NA | |  | 4 |
| *Euphorbia peplus* | | herb | IN | Eurasia | Paleartic | | Cosmopolitan | 2 |
| *Evodianthus funifer* | | herb | NAT | NA | NA | |  | 4 |
| *Faramea exemplaris* | | shrub | NAT | NA | NA | |  | 1 |
| *Festuca andicola* | | herb | NAT | NA | NA | |  | 78 |
| *Festuca asplundii* | | herb | NAT | NA | NA | |  | 8 |
| *Festuca subulifolia* | | herb | NAT | NA | NA | |  | 67 |
| *Ficus brevibracteata* | | treelet | NAT | NA | NA | |  | 3 |
| *Ficus coerulescens* | | tree | NAT | NA | NA | |  | 2 |
| *Fimbristylis dichotoma* | | herb | NAT | NA | NA | |  | 2 |
| *Floscopa peruviana* | | shrub | NAT | NA | NA | |  | 4 |
| *Fragaria vesca* | | herb | IN | Eurasia | Paleartic | | Temperate | 26 |
| *Freziera reticulata* | | shrub | NAT | NA | NA | |  | 1 |
| *Fuchsia canescens* | | shrub | NAT | NA | NA | |  | 23 |
| *Fuchsia macrostigma* | | shrub | NAT | NA | NA | |  | 1 |
| *Fuchsia scabriuscula* | | shrub | NAT | NA | NA | |  | 39 |
| *Galinsoga parviflora* | | herb | NAT | NA | NA | |  | 66 |
| *Galinsoga quadriradiata* | | herb | NAT | NA | NA | |  | 19 |
| *Galium aparine* | | herb | NAT | NA | NA | |  | 1 |
| *Galium canescens* | | herb | NAT | NA | NA | |  | 20 |
| *Galium corymbosum* | | herb | NAT | NA | NA | |  | 24 |
| *Galium hypocarpium* | | herb | NAT | NA | NA | |  | 29 |
| *Galium pseudotriflorum* | | herb | NAT | NA | NA | |  | 7 |
| *Gamochaeta americana* | | herb | NAT | NA | NA | |  | 206 |
| *Gamochaeta purpurea* | | herb | NAT | NA | NA | |  | 10 |
| *Gaultheria foliolosa* | | shrub | NAT | NA | NA | |  | 1 |
| *Gaultheria glomerata* | | shrub | NAT | NA | NA | |  | 16 |
| *Gentiana sedifolia* | | herb | NAT | NA | NA | |  | 7 |
| *Gentianella limoselloides* | | herb | E | NA | NA | |  | 9 |
| *Gentianella rapunculoides* | | herb | NAT | NA | NA | |  | 11 |
| *Geranium antisanae* | | herb | E | NA | NA | |  | 10 |
| *Geranium chilloense* | | herb | NAT | NA | NA | |  | 21 |
| *Geranium diffusum* | | herb | NAT | NA | NA | |  | 23 |
| *Geranium humboldtii* | | herb | NAT | NA | NA | |  | 11 |
| *Geranium* cf. *knuthianum* | | herb | NAT | NA | NA | |  | 5 |
| *Geranium maniculatum* | | herb | NAT | NA | NA | |  | 19 |
| *Geranium multipartitum* | | herb | NAT | NA | NA | |  | 8 |
| *Geranium pseudodiffusum* | | herb | NAT | NA | NA | |  | 5 |
| *Geranium reptans* | | herb | NAT | NA | NA | |  | 48 |
| *Geranium sibbaldioides* | | herb | NAT | NA | NA | |  | 2 |
| *Geranium stramineum* | | herb | NAT | NA | NA | |  | 21 |
| *Geum peruvianum* | | herb | NAT | NA | NA | |  | 1 |
| *Glossoloma tetragonoides* | | shrub | E | NA | NA | |  | 20 |
| *Gloxinia dodsonii* | | herb | NAT | NA | NA | |  | 6 |
| *Gnaphalium antennarioides* | | herb | NAT | NA | NA | |  | 79 |
| *Gnaphalium dombeyanum* | | herb | NAT | NA | NA | |  | 7 |
| *Gnaphalium purpureum* | | herb | NAT | NA | NA | |  | 53 |
| *Gnaphalium* cf. *tenue* | | herb | NAT | NA | NA | |  | 3 |
| *Gonzalagunia bunchosioides* | | shrub | NAT | NA | NA | |  | 7 |
| *Gonzalagunia sororia* | | shrub | NAT | NA | NA | |  | 1 |
| *Grosvenoria rimbachii* | | shrub | E | NA | NA | |  | 14 |
| *Guatteria asplundiana* | | tree | NAT | NA | NA | |  | 5 |
| *Guettarda crispiflora* | | shrub | NAT | NA | NA | |  | 3 |
| *Gunnera brephogea* | | shrub | NAT | NA | NA | |  | 36 |
| *Gunnera magellanica* | | herb | NAT | NA | NA | |  | 48 |
| *Gurania pedata* | | liana | NAT | NA | NA | |  | 4 |
| *Gurania rhizantha* | | liana | NAT | NA | NA | |  | 3 |
| *Gurania tubulosa* | | liana | NAT | NA | NA | |  | 2 |
| *Gynerium sagittatum* | | herb | NAT | NA | NA | |  | 11 |
| *Gynoxys acostae* | | shrub | E | NA | NA | |  | 9 |
| *Gynoxys buxifolia* | | shrub | NAT | NA | NA | |  | 1 |
| *Halenia longicaulis* | | herb | E | NA | NA | |  | 2 |
| *Halenia weddelliana* | | herb | NAT | NA | NA | |  | 8 |
| *Hedychium coronarium* | | herb | IN | Asia | Paleartic | | Temperate | 14 |
| *Hedyosmum angustifolium* | | treelet | NAT | NA | NA | |  | 3 |
| *Hedyosmum anisodorum* | | treelet | NAT | NA | NA | |  | 2 |
| *Hedyosmum cuatrecazanum* | | treelet | NAT | NA | NA | |  | 7 |
| *Hedyosmum racemosum* | | treelet | NAT | NA | NA | |  | 1 |
| *Hedyosmum sprucei* | | treelet | NAT | NA | NA | |  | 9 |
| *Heliconia* cf. *aemygdiana* | | herb | NAT | NA | NA | |  | 4 |
| *Heliconia burleana* | | herb | NAT | NA | NA | |  | 1 |
| *Heliconia* aff. *hirsuta* | | herb | NAT | NA | NA | |  | 7 |
| *Heliocarpus americanus* | | tree | NAT | NA | NA | |  | 6 |
| *Heliopsis oppositifolia* | | herb | NAT | NA | NA | |  | 14 |
| *Heliotropium rufipilum* | | herb | NAT | NA | NA | |  | 3 |
| *Heppiella repens* | | herb | NAT | NA | NA | |  | 24 |
| *Hesperomeles ferruginea* | | treelet | NAT | NA | NA | |  | 1 |
| *Hesperomeles obtusifolia* | | treelet | NAT | NA | NA | |  | 27 |
| *Hieracium frigidum* | | herb | NAT | NA | NA | |  | 44 |
| *Hieronyma alchorneoides* | | tree | NAT | NA | NA | |  | 6 |
| *Histiopteris incisa* | | ferm | NAT | NA | NA | |  | 2 |
| *Holcus lanatus* | | herb | IN | Europe | Paleartic | | Temperate | 175 |
| *Hydrocotyle alchemilloides* | | herb | NAT | NA | NA | |  | 1 |
| *Hydrocotyle bonplandii* | | herb | NAT | NA | NA | |  | 98 |
| *Hydrocotyle hitchcockii* | | herb | NAT | NA | NA | |  | 23 |
| *Hydrocotyle palmata* | | herb | NAT | NA | NA | |  | 14 |
| *Hydrocotyle pusilla* | | herb | NAT | NA | NA | |  | 16 |
| *Hydrocotyle umbellata* | | herb | NAT | NA | NA | |  | 1 |
| *Hypericum brevistylum* | | herb | NAT | NA | NA | |  | 3 |
| *Hypericum lancioides* | | shrub | NAT | NA | NA | |  | 25 |
| *Hypericum laricifolium* | | shrub | NAT | NA | NA | |  | 9 |
| *Hypericum silenoides* | | herb | NAT | NA | NA | |  | 5 |
| *Hypochaeris sessiliflora* | | herb | NAT | NA | NA | |  | 8 |
| *Hypochaeris sonchoides* | | herb | E | NA | NA | |  | 12 |
| *Hypolepis bogotensis* | | ferm | NAT | NA | NA | |  | 3 |
| *Hyptis mutabilis* | | herb | NAT | NA | NA | |  | 2 |
| *Hyptis obtusiflora* | | herb | NAT | NA | NA | |  | 21 |
| *Ichnanthus nemorosus* | | herb | NAT | NA | NA | |  | 2 |
| *Ichnanthus pallens* | | herb | NAT | NA | NA | |  | 17 |
| *Inga densiflora* | | tree | NAT | NA | NA | |  | 10 |
| *Inga edulis* | | tree | NAT | NA | NA | |  | 24 |
| *Inga marginata* | | tree | NAT | NA | NA | |  | 13 |
| *Inga nobilis* | | tree | NAT | NA | NA | |  | 1 |
| *Inga oerstediana* | | tree | NAT | NA | NA | |  | 2 |
| *Inga ruiziana* | | tree | NAT | NA | NA | |  | 1 |
| *Inga suaveolens* | | tree | NAT | NA | NA | |  | 1 |
| *Inga* cf. *tenuistipula* | | tree | NAT | NA | NA | |  | 2 |
| *Ipomoea ophiodes* | | liana | NAT | NA | NA | |  | 3 |
| *Iresine diffusa* | | herb | NAT | NA | NA | |  | 17 |
| *Isertia laevis* | | tree | NAT | NA | NA | |  | 4 |
| *Jamesonia goudotii* | | ferm | NAT | NA | NA | |  | 6 |
| *Juncus bufonius* | | herb | NAT | NA | NA | |  | 53 |
| *Juncus effusus* | | herb | NAT | NA | NA | |  | 2 |
| *Juncus microcephalus* | | herb | NAT | NA | NA | |  | 12 |
| *Jungia coarctata* | | liana | NAT | NA | NA | |  | 9 |
| *Kyllinga pumila* | | herb | NAT | NA | NA | |  | 40 |
| *Lachemilla aphanoides* | | herb | NAT | NA | NA | |  | 51 |
| *Lachemilla* cf. *fulvescens* | | herb | NAT | NA | NA | |  | 3 |
| *Lachemilla hispidula* | | herb | NAT | NA | NA | |  | 14 |
| *Lachemilla* cf. *jamesonii* | | herb | E | NA | NA | |  | 17 |
| *Lachemilla mandoniana* | | herb | NAT | NA | NA | |  | 7 |
| *Lachemilla nivalis* | | herb | NAT | NA | NA | |  | 8 |
| *Lachemilla orbiculata* | | herb | NAT | NA | NA | |  | 103 |
| *Lachemilla pectinata* | | herb | NAT | NA | NA | |  | 8 |
| *Lachemilla perryana* | | herb | NAT | NA | NA | |  | 2 |
| *Lachemilla vulcanica* | | herb | NAT | NA | NA | |  | 9 |
| *Lantana camara* | | shrub | IN | Tropical America | Neotropical | | Tropical | 7 |
| *Lasiacis divaricata* | | herb | NAT | NA | NA | |  | 1 |
| *Lasiacis sorghoidea* | | herb | NAT | NA | NA | |  | 18 |
| *Lasiocephalus involucratus* | | shrub | E | NA | NA | |  | 11 |
| *Lasiocephalus patens* | | herb | NAT | NA | NA | |  | 5 |
| *Leandra chaetodon* | | shrub | NAT | NA | NA | |  | 2 |
| *Lepidium bipinnatifidum* | | herb | NAT | NA | NA | |  | 14 |
| *Liabum kingii* | | shrub | E | NA | NA | |  | 22 |
| *Licaria guianensis* | | tree | NAT | NA | NA | |  | 1 |
| *Lophosoria quadripinnata* | | ferm | NAT | NA | NA | |  | 28 |
| *Loricaria thuyoides* | | shrub | NAT | NA | NA | |  | 16 |
| *Lupinus microphyllus* | | herb | NAT | NA | NA | |  | 7 |
| *Lupinus pubescens* | | shrub | NAT | NA | NA | |  | 17 |
| *Luzula gigantea* | | herb | NAT | NA | NA | |  | 45 |
| *Lycianthes radiata* | | shrub | NAT | NA | NA | |  | 1 |
| *Macleania bullata* | | shrub | NAT | NA | NA | |  | 4 |
| *Macleania rupestris* | | shrub | NAT | NA | NA | |  | 2 |
| *Macrocarpaea pringleana* | | shrub | NAT | NA | NA | |  | 14 |
| *Macrothelypteris torresiana* | | ferm | IN | Asia, Africa | Paleotropical | | Tropical | 25 |
| *Malva pusilla* | | herb | IN | Eurasia, north Africa | Paleartic | | Temperate | 1 |
| *Manettia cordifolia* | | liana | NAT | NA | NA | |  | 9 |
| *Manettia lobbii* | | shrub | NAT | NA | NA | |  | 1 |
| *Mecardonia procumbens* | | herb | NAT | NA | NA | |  | 1 |
| *Melissa officinalis* | | herb | IN | Europe, Mediterranean | Paleartic | | Temperate | 9 |
| *Melpomene pseudonutans* | | ferm | NAT | NA | NA | |  | 3 |
| *Mendoncia brenesii* | | liana | NAT | NA | NA | |  | 4 |
| *Mendoncia glabra* | | liana | NAT | NA | NA | |  | 14 |
| *Mendoncia lindavii* | | liana | NAT | NA | NA | |  | 7 |
| *Mendoncia sericea* | | liana | NAT | NA | NA | |  | 1 |
| *Meriania finicola* | | tree | NAT | NA | NA | |  | 2 |
| *Miconia affinis* | | treelet | NAT | NA | NA | |  | 21 |
| *Miconia aggregata* | | shrub | NAT | NA | NA | |  | 2 |
| *Miconia asclepiadea* | | shrub | NAT | NA | NA | |  | 15 |
| *Miconia astroplocama* | | shrub | NAT | NA | NA | |  | 1 |
| *Miconia bracteolata* | | shrub | NAT | NA | NA | |  | 1 |
| *Miconia brevitheca* | | tree | E | NA | NA | |  | 21 |
| *Miconia calvescens* | | tree | NAT | NA | NA | |  | 1 |
| *Miconia cercophora* | | shrub | E | NA | NA | |  | 9 |
| *Miconia clathrantha* | | tree | NAT | NA | NA | |  | 1 |
| *Miconia crocea* | | shrub | NAT | NA | NA | |  | 9 |
| *Miconia dolichorrhyncha* | | treelet | NAT | NA | NA | |  | 3 |
| *Miconia elata* | | tree | NAT | NA | NA | |  | 1 |
| *Miconia floribunda* | | treelet | NAT | NA | NA | |  | 1 |
| *Miconia gibba* | | treelet | E | NA | NA | |  | 3 |
| *Miconia grandifolia* | | shrub | NAT | NA | NA | |  | 5 |
| *Miconia manicata* | | shrub | NAT | NA | NA | |  | 15 |
| *Miconia multispicata* | | shrub | NAT | NA | NA | |  | 5 |
| *Miconia pseudocentrophora* | | shrub | NAT | NA | NA | |  | 17 |
| *Miconia salicifolia* | | shrub | NAT | NA | NA | |  | 8 |
| *Mikania banisteriae* | | liana | NAT | NA | NA | |  | 8 |
| *Mikania cordifolia* | | liana | NAT | NA | NA | |  | 22 |
| *Mikania cuzcoensis* | | liana | NAT | NA | NA | |  | 1 |
| *Mikania decora* | | liana | NAT | NA | NA | |  | 6 |
| *Mikania granulata* | | liana | NAT | NA | NA | |  | 6 |
| *Mikania guaco* | | liana | NAT | NA | NA | |  | 1 |
| *Mikania hitchcockii* | | liana | E | NA | NA | |  | 8 |
| *Mikania hookeriana* | | liana | NAT | NA | NA | |  | 5 |
| *Mikania iodotricha* | | liana | E | NA | NA | |  | 5 |
| *Mikania leiostachya* | | liana | NAT | NA | NA | |  | 13 |
| *Mikania micrantha* | | liana | NAT | NA | NA | |  | 14 |
| *Mikania nigropunctulata* | | liana | NAT | NA | NA | |  | 1 |
| *Mikania parviflora* | | liana | NAT | NA | NA | |  | 1 |
| *Mimosa polydactyla* | | herb | NAT | NA | NA | |  | 5 |
| *Minthostachys mollis* | | herb | NAT | NA | NA | |  | 14 |
| *Monnina* aff. *cestrifolia* | | shrub | E | NA | NA | |  | 4 |
| *Monnina crassifolia* | | shrub | NAT | NA | NA | |  | 6 |
| *Monnina marginata* | | shrub | NAT | NA | NA | |  | 5 |
| *Monochaetum lineatum* | | shrub | NAT | NA | NA | |  | 56 |
| *Monticalia arbutifolia* | | shrub | NAT | NA | NA | |  | 4 |
| *Monticalia peruviana* | | shrub | NAT | NA | NA | |  | 3 |
| *Morella pubescens* | | tree | NAT | NA | NA | |  | 12 |
| *Morus insignis* | | treelet | NAT | NA | NA | |  | 1 |
| *Muehlenbeckia tamnifolia* | | shrub | NAT | NA | NA | |  | 20 |
| *Muehlenbeckia volcanica* | | shrub | NAT | NA | NA | |  | 33 |
| *Munnozia jussieui* | | shrub | NAT | NA | NA | |  | 1 |
| *Munnozia senecionidis* | | shrub | NAT | NA | NA | |  | 66 |
| *Myrciaria floribunda* | | treelet | NAT | NA | NA | |  | 1 |
| *Myrsine guianensis* | | tree | NAT | NA | NA | |  | 1 |
| *Nasturtium officinale* | | herb | IN | Eurasia, north Africa | Paleartic | | Temperate | 38 |
| *Nectandra acutifolia* | | tree | NAT | NA | NA | |  | 8 |
| *Nectandra membranacea* | | tree | NAT | NA | NA | |  | 1 |
| *Nectandra parviflora* | | tree | E | NA | NA | |  | 3 |
| *Neea parviflora* | | treelet | NAT | NA | NA | |  | 1 |
| *Nertera granadensis* | | herb | NAT | NA | NA | |  | 101 |
| *Niphidium crassifolium* | | ferm | NAT | NA | NA | |  | 10 |
| *Niphogeton dissecta* | | herb | NAT | NA | NA | |  | 40 |
| *Niphogeton ternata* | | herb | NAT | NA | NA | |  | 5 |
| *Notopleura madida* | | shrub | E | NA | NA | |  | 2 |
| *Ochroma pyramidale* | | tree | NAT | NA | NA | |  | 3 |
| *Ocotea atirrensis* | | treelet | NAT | NA | NA | |  | 3 |
| *Ocotea bofo* | | tree | NAT | NA | NA | |  | 2 |
| *Ocotea cernua* | | tree | NAT | NA | NA | |  | 2 |
| *Oenothera rosea* | | herb | NAT | NA | NA | |  | 3 |
| *Ophioglossum crotalophoroides* | | ferm | NAT | NA | NA | |  | 1 |
| *Oreomyrrhis andicola* | | herb | NAT | NA | NA | |  | 57 |
| *Oreopanax ecuadorensis* | | tree | E | NA | NA | |  | 18 |
| *Otholobium mexicanum* | | shrub | NAT | NA | NA | |  | 5 |
| *Otoba parvifolia* | | tree | NAT | NA | NA | |  | 4 |
| *Oxalis bisfracta* | | herb | NAT | NA | NA | |  | 105 |
| *Oxalis integra* | | herb | NAT | NA | NA | |  | 72 |
| *Oxalis lotoides* | | herb | NAT | NA | NA | |  | 29 |
| *Oxalis mollis* | | herb | NAT | NA | NA | |  | 9 |
| *Oxalis spiralis* | | herb | NAT | NA | NA | |  | 10 |
| *Palicourea candida* | | shrub | E | NA | NA | |  | 8 |
| *Palicourea lasiantha* | | shrub | NAT | NA | NA | |  | 10 |
| *Panicum laxum* | | herb | NAT | NA | NA | |  | 28 |
| *Panicum trichanthum* | | herb | NAT | NA | NA | |  | 2 |
| *Paspalum conjugatum* | | herb | NAT | NA | NA | |  | 11 |
| *Paspalum humboldtianum* | | herb | NAT | NA | NA | |  | 5 |
| *Paspalum paniculatum* | | herb | NAT | NA | NA | |  | 18 |
| *Paspalum penicillatum* | | herb | NAT | NA | NA | |  | 12 |
| *Paspalum pilgerianum* | | herb | NAT | NA | NA | |  | 1 |
| *Paspalum plicatulum* | | herb | NAT | NA | NA | |  | 2 |
| *Paspalum reclinatum* | | herb | NAT | NA | NA | |  | 9 |
| *Paspalum saccharoides* | | herb | NAT | NA | NA | |  | 9 |
| *Passiflora alnifolia* | | liana | NAT | NA | NA | |  | 2 |
| *Passiflora* cf. *andina* | | liana | NAT | NA | NA | |  | 3 |
| *Passiflora* cf. *cumbalensis* | | liana | NAT | NA | NA | |  | 1 |
| *Passiflora ligularis* | | liana | NAT | NA | NA | |  | 4 |
| *Passiflora tryphostemmatoides* | | liana | NAT | NA | NA | |  | 7 |
| *Paullinia dasystachya* | | liana | NAT | NA | NA | |  | 1 |
| *Paullinia serjaniifolia* | | liana | NAT | NA | NA | |  | 1 |
| *Pennisetum bambusiforme* | | herb | NAT | NA | NA | |  | 78 |
| *Pennisetum clandestinum* | | herb | IN | Africa | Paleotropical | | Tropical | 180 |
| *Pentacalia andicola* | | shrub | NAT | NA | NA | |  | 4 |
| *Pentacalia disciformis* | | shrub | NAT | NA | NA | |  | 4 |
| *Pentacalia vaccinioides* | | shrub | NAT | NA | NA | |  | 3 |
| *Pentagonia amazonica* | | tree | NAT | NA | NA | |  | 1 |
| *Pentagonia macrophylla* | | treelet | NAT | NA | NA | |  | 1 |
| *Peperomia alwynii* | | herb | NAT | NA | NA | |  | 5 |
| *Peperomia caespitosa* | | herb | NAT | NA | NA | |  | 6 |
| *Peperomia eburnea* | | herb | NAT | NA | NA | |  | 7 |
| *Peperomia galioides* | | herb | NAT | NA | NA | |  | 1 |
| *Peperomia glabella* | | herb | NAT | NA | NA | |  | 11 |
| *Peperomia hartwegiana* | | herb | NAT | NA | NA | |  | 4 |
| *Peperomia hispidula* | | herb | NAT | NA | NA | |  | 22 |
| *Peperomia hispiduliformis* | | herb | NAT | NA | NA | |  | 11 |
| *Peperomia peltoidea* | | herb | NAT | NA | NA | |  | 5 |
| *Peperomia rotundata* | | herb | NAT | NA | NA | |  | 23 |
| *Peperomia sachatzinzumba* | | herb | NAT | NA | NA | |  | 2 |
| *Pernettya prostrata* | | shrub | NAT | NA | NA | |  | 57 |
| *Persea mutisii* | | shrub | NAT | NA | NA | |  | 2 |
| *Petroselinum crispum* | | herb | IN | Mediterranean | Paleartic | | Temperate | 10 |
| *Phenax rugosus* | | shrub | NAT | NA | NA | |  | 11 |
| *Philodendron campii* | | herb | NAT | NA | NA | |  | 2 |
| *Philodendron colombianum* | | herb | NAT | NA | NA | |  | 8 |
| *Philodendron mamei* | | herb | NAT | NA | NA | |  | 10 |
| *Philodendron pteropus* | | herb | NAT | NA | NA | |  | 4 |
| *Phyllanthus brasiliensis* | | shrub | NAT | NA | NA | |  | 7 |
| *Phyllanthus caroliniensis* | | herb | NAT | NA | NA | |  | 6 |
| *Phytolacca bogotensis* | | herb | NAT | NA | NA | |  | 10 |
| *Phytolacca rivinoides* | | herb | NAT | NA | NA | |  | 16 |
| *Pilea antioquensis* | | shrub | NAT | NA | NA | |  | 11 |
| *Pilea arguta* | | herb | NAT | NA | NA | |  | 5 |
| *Pilea attenuata* | | herb | E | NA | NA | |  | 3 |
| *Pilea microphylla* | | herb | NAT | NA | NA | |  | 12 |
| *Pilea mutisiana* | | herb | NAT | NA | NA | |  | 4 |
| *Pilea napoana* | | shrub | E | NA | NA | |  | 4 |
| *Pilea trichosanthes* | | herb | E | NA | NA | |  | 15 |
| *Piper aduncum* | | shrub | NAT | NA | NA | |  | 25 |
| *Piper albozonatum* | | shrub | NAT | NA | NA | |  | 2 |
| *Piper barbatum* | | shrub | NAT | NA | NA | |  | 2 |
| *Piper* cf. *bullosum* | | shrub | NAT | NA | NA | |  | 3 |
| *Piper carpunya* | | shrub | NAT | NA | NA | |  | 3 |
| *Piper crassinervium* | | shrub | NAT | NA | NA | |  | 6 |
| *Piper hispidum* | | shrub | NAT | NA | NA | |  | 3 |
| *Piper immutatum* | | shrub | NAT | NA | NA | |  | 4 |
| *Piper lacunosum* | | shrub | NAT | NA | NA | |  | 14 |
| *Piper lanceifolium* | | shrub | NAT | NA | NA | |  | 20 |
| *Piper* cf. *lanceolatum* | | shrub | NAT | NA | NA | |  | 1 |
| *Piper lehmannianum* | | shrub | NAT | NA | NA | |  | 1 |
| *Piper lineatum* | | shrub | NAT | NA | NA | |  | 14 |
| *Piper musteum* | | shrub | NAT | NA | NA | |  | 4 |
| *Piper* cf. *peltatum* | | shrub | NAT | NA | NA | |  | 2 |
| *Piper soledadense* | | shrub | NAT | NA | NA | |  | 1 |
| *Piper veraguense* | | shrub | NAT | NA | NA | |  | 1 |
| *Piptocoma discolor* | | treelet | NAT | NA | NA | |  | 11 |
| *Pityrogramma calomelanos* | | ferm | NAT | NA | NA | |  | 4 |
| *Pityrogramma tartarea* | | ferm | NAT | NA | NA | |  | 10 |
| *Plagiocheilus solivaeformis* | | herb | NAT | NA | NA | |  | 28 |
| *Plantago australis* | | herb | NAT | NA | NA | |  | 94 |
| *Plantago lanceolata* | | herb | IN | Eurasia, north America | Holarctic | | Temperate | 13 |
| *Plantago linearis* | | herb | NAT | NA | NA | |  | 8 |
| *Plantago major* | | herb | IN | Eurasia, north America | Holarctic | | Temperate | 45 |
| *Plantago rigida* | | herb | NAT | NA | NA | |  | 3 |
| *Poa aequatoriensis* | | herb | NAT | NA | NA | |  | 2 |
| *Poa annua* | | herb | IN | Eurasia | Paleartic | | Temperate | 59 |
| *Poa mulalensis* | | herb | E | NA | NA | |  | 2 |
| *Poa paramoensis* | | herb | E | NA | NA | |  | 7 |
| *Poa pauciflora* | | herb | NAT | NA | NA | |  | 7 |
| *Poa subspicata* | | herb | NAT | NA | NA | |  | 4 |
| *Polybotrya polybotryoides* | | ferm | NAT | NA | NA | |  | 8 |
| *Polygala paniculata* | | herb | NAT | NA | NA | |  | 1 |
| *Polygonum hydropiperoides* | | herb | NAT | NA | NA | |  | 3 |
| *Polygonum nepalense* | | herb | IN | Asia | Paleartic | | Temperate | 2 |
| *Polygonum punctatum* | | herb | NAT | NA | NA | |  | 15 |
| *Polypodium mindense* | | ferm | E | NA | NA | |  | 1 |
| *Polypodium* cf. *monosorum* | | ferm | NAT | NA | NA | |  | 43 |
| *Polypodium remotum* | | ferm | NAT | NA | NA | |  | 4 |
| *Polypodium sessilifolium* | | ferm | NAT | NA | NA | |  | 1 |
| *Polypogon interruptus* | | herb | NAT | NA | NA | |  | 15 |
| *Polystichum lehmannii* | | ferm | NAT | NA | NA | |  | 9 |
| *Polystichum orbiculatum* | | ferm | NAT | NA | NA | |  | 12 |
| *Pourouma guianensis* | | tree | NAT | NA | NA | |  | 8 |
| *Protium amazonicum* | | tree | NAT | NA | NA | |  | 1 |
| *Prunella vulgaris* | | herb | IN | Eurasia | Paleartic | | Cosmopolitan | 67 |
| *Prunus serotina* | | tree | NAT | NA | NA | |  | 2 |
| *Psammisia aberrans* | | shrub | NAT | NA | NA | |  | 17 |
| *Psammisia ferruginea* | | shrub | NAT | NA | NA | |  | 11 |
| *Psammisia sodiroi* | | shrub | NAT | NA | NA | |  | 16 |
| *Pseudoelephantopus spiralis* | | herb | NAT | NA | NA | |  | 5 |
| *Psidium guajava* | | treelet | NAT | NA | NA | |  | 10 |
| *Psychotria acuminata* | | shrub | NAT | NA | NA | |  | 12 |
| *Psychotria fusiformis* | | shrub | E | NA | NA | |  | 6 |
| *Psychotria poeppigiana* | | shrub | NAT | NA | NA | |  | 9 |
| *Psychotria tinctoria* | | shrub | NAT | NA | NA | |  | 2 |
| *Pteris muricata* | | ferm | NAT | NA | NA | |  | 10 |
| *Pycreus bipartitus* | | shrub | NAT | NA | NA | |  | 18 |
| *Ranunculus limoselloides* | | herb | NAT | NA | NA | |  | 1 |
| *Ranunculus peruvianus* | | herb | NAT | NA | NA | |  | 10 |
| *Ranunculus praemorsus* | | herb | NAT | NA | NA | |  | 16 |
| *Rhynchospora blepharophora* | | herb | NAT | NA | NA | |  | 5 |
| *Rhynchospora dissitiflora* | | herb | NAT | NA | NA | |  | 71 |
| *Rhynchospora ruiziana* | | herb | NAT | NA | NA | |  | 18 |
| *Ribes ecuadorense* | | shrub | NAT | NA | NA | |  | 13 |
| *Ribes hirtum* | | shrub | NAT | NA | NA | |  | 1 |
| *Rorippa* cf. *bonariensis* | | herb | NAT | NA | NA | |  | 2 |
| *Rubus adenotrichos* | | liana | NAT | NA | NA | |  | 32 |
| *Rubus* cf. *boliviensis* | | shrub | NAT | NA | NA | |  | 5 |
| *Rubus glabratus* | | shrub | NAT | NA | NA | |  | 24 |
| *Rubus glaucus* | | shrub | NAT | NA | NA | |  | 8 |
| *Rubus niveus* | | shrub | IN | Asia | Paleartic | | Temperate | 5 |
| *Rubus nubigenus* | | liana | NAT | NA | NA | |  | 13 |
| *Rubus urticifolius* | | shrub | NAT | NA | NA | |  | 14 |
| *Rumex acetosella* | | herb | IN | Eurasia | Paleartic | | Temperate | 46 |
| *Rumex crispus* | | herb | IN | Europe, west Asia | Paleartic | | Cosmopolitan | 9 |
| *Rytidostylis carthagenensis* | | liana | NAT | NA | NA | |  | 9 |
| *Rytidostylis trianaei* | | liana | NAT | NA | NA | |  | 6 |
| *Sagina procumbens* | | herb | IN | Eurasia, north Africa | Paleartic | | Temperate | 15 |
| *Salvia macrophylla* | | shrub | NAT | NA | NA | |  | 7 |
| *Salvia pichinchensis* | | shrub | NAT | NA | NA | |  | 2 |
| *Salvia quitensis* | | shrub | E | NA | NA | |  | 4 |
| *Salvia scutellarioides* | | herb | NAT | NA | NA | |  | 5 |
| *Saurauia adenodonta* | | treelet | E | NA | NA | |  | 9 |
| *Saurauia aequatoriensis* | | shrub | E | NA | NA | |  | 6 |
| *Saurauia bullosa* | | shrub | NAT | NA | NA | |  | 2 |
| *Saurauia herthae* | | treelet | E | NA | NA | |  | 6 |
| *Saurauia isoxanthotricha* | | shrub | NAT | NA | NA | |  | 5 |
| *Saurauia laxiflora* | | treelet | E | NA | NA | |  | 5 |
| *Saurauia prainiana* | | shrub | NAT | NA | NA | |  | 12 |
| *Saurauia rubrisepala* | | shrub | E | NA | NA | |  | 2 |
| *Schefflera diplodactyla* | | treelet | NAT | NA | NA | |  | 3 |
| *Schefflera morototoni* | | tree | NAT | NA | NA | |  | 2 |
| *Schizolobium parahyba* | | tree | NAT | NA | NA | |  | 1 |
| *Selaginella diffusa* | | herb | NAT | NA | NA | |  | 33 |
| *Selaginella* cf. *flagellata* | | herb | NAT | NA | NA | |  | 3 |
| *Senecio culcitioides* | | herb | NAT | NA | NA | |  | 12 |
| *Senecio tephrosioides* | | herb | NAT | NA | NA | |  | 3 |
| *Senefeldera inclinata* | | tree | NAT | NA | NA | |  | 1 |
| *Senna ruiziana* | | tree | NAT | NA | NA | |  | 11 |
| *Serpocaulon fraxinifolium* | | ferm | NAT | NA | NA | |  | 6 |
| *Serpocaulon lasiopus* | | ferm | NAT | NA | NA | |  | 12 |
| *Serpocaulon levigatum* | | ferm | NAT | NA | NA | |  | 3 |
| *Sessea crassivenosa* | | shrub | NAT | NA | NA | |  | 6 |
| *Sessea vestita* | | shrub | NAT | NA | NA | |  | 8 |
| *Setaria sphacelata* | | herb | IN | Africa | Paleotropical | | Tropical | 12 |
| *Sibthorpia repens* | | herb | NAT | NA | NA | |  | 36 |
| *Sida abutifolia* | | herb | NAT | NA | NA | |  | 1 |
| *Silene* cf. *gallica* | | herb | IN | Mediterranean | Paleartic | | Temperate | 9 |
| *Siparuna aspera* | | shrub | NAT | NA | NA | |  | 2 |
| *Siparuna harlingii* | | shrub | NAT | NA | NA | |  | 1 |
| *Siparuna lepidota* | | shrub | NAT | NA | NA | |  | 1 |
| *Siphocampylus lucidus* | | shrub | E | NA | NA | |  | 1 |
| *Sisyrinchium chilense* | | herb | NAT | NA | NA | |  | 16 |
| *Smilax poeppigii* | | liana | NAT | NA | NA | |  | 1 |
| *Solanum abitaguense* | | shrub | NAT | NA | NA | |  | 1 |
| *Solanum americanum* | | herb | NAT | NA | NA | |  | 25 |
| *Solanum anceps* | | shrub | NAT | NA | NA | |  | 1 |
| *Solanum asperolanatum* | | shrub | NAT | NA | NA | |  | 19 |
| *Solanum* cf. *aturense* | | liana | NAT | NA | NA | |  | 2 |
| *Solanum barbulatum* | | shrub | NAT | NA | NA | |  | 2 |
| *Solanum colombianum* | | shrub | NAT | NA | NA | |  | 8 |
| *Solanum hypermegethes* | | treelet | E | NA | NA | |  | 1 |
| *Solanum juglandifolium* | | shrub | NAT | NA | NA | |  | 2 |
| *Solanum lepidotum* | | shrub | NAT | NA | NA | |  | 2 |
| *Solanum nutans* | | shrub | NAT | NA | NA | |  | 5 |
| *Solanum oblongifolium* | | shrub | NAT | NA | NA | |  | 18 |
| *Solanum ovalifolium* | | shrub | NAT | NA | NA | |  | 2 |
| *Solanum rugosum* | | shrub | NAT | NA | NA | |  | 1 |
| *Solanum stenophyllum* | | shrub | NAT | NA | NA | |  | 1 |
| *Solanum trachycyphum* | | shrub | NAT | NA | NA | |  | 1 |
| *Sonchus asper* | | herb | IN | Eurasia, Africa | Paleartic | | Temperate | 7 |
| *Sonchus oleraceus* | | herb | IN | Eurasia, north Africa | Paleartic | | Temperate | 44 |
| *Sorocea pubivena* | | treelet | NAT | NA | NA | |  | 1 |
| *Sporobolus indicus* | | herb | NAT | NA | NA | |  | 31 |
| *Stachys elliptica* | | herb | E | NA | NA | |  | 9 |
| *Stellaria media* | | herb | NAT | NA | NA | |  | 59 |
| *Stellaria recurvata* | | herb | E | NA | NA | |  | 47 |
| *Stellaria serpyllifolia* | | herb | NAT | NA | NA | |  | 36 |
| *Sticherus bifidus* | | ferm | NAT | NA | NA | |  | 31 |
| *Stigmaphyllon alternans* | | liana | NAT | NA | NA | |  | 3 |
| *Stigmaphyllon bogotense* | | liana | NAT | NA | NA | |  | 2 |
| *Syngonium podophyllum* | | herb | NAT | NA | NA | |  | 4 |
| *Taraxacum officinale* | | herb | IN | Eurasia | Paleartic | | Temperate | 157 |
| *Teliostachya lanceolata* | | herb | NAT | NA | NA | |  | 4 |
| *Tessaria integrifolia* | | treelet | NAT | NA | NA | |  | 2 |
| *Tetrathylacium macrophyllum* | | shrub | NAT | NA | NA | |  | 4 |
| *Tetrorchidium andinum* | | shrub | NAT | NA | NA | |  | 1 |
| *Tetrorchidium euryphyllum* | | shrub | NAT | NA | NA | |  | 4 |
| *Thalictrum podocarpum* | | herb | NAT | NA | NA | |  | 4 |
| *Thelypteris* cf. *amphioxypteris* | | ferm | NAT | NA | NA | |  | 1 |
| *Thelypteris cheilanthoides* | | ferm | NAT | NA | NA | |  | 26 |
| *Thelypteris concinna* | | ferm | NAT | NA | NA | |  | 1 |
| *Thelypteris curta* | | ferm | NAT | NA | NA | |  | 1 |
| *Thelypteris euchlora* | | ferm | NAT | NA | NA | |  | 1 |
| *Thelypteris glandulosa* | | ferm | NAT | NA | NA | |  | 14 |
| *Thelypteris opulenta* | | ferm | IN | Tropical, subtropical | Paleotropical | | Tropical | 117 |
| *Thelypteris pachyrhachis* | | ferm | NAT | NA | NA | |  | 14 |
| *Thelypteris patens* | | ferm | NAT | NA | NA | |  | 50 |
| *Thelypteris pilosohispida* | | ferm | NAT | NA | NA | |  | 13 |
| *Thelypteris pteroidea* | | ferm | NAT | NA | NA | |  | 2 |
| *Thelypteris* cf. *rigescens* | | ferm | NAT | NA | NA | |  | 6 |
| *Tibouchina lepidota* | | treelet | NAT | NA | NA | |  | 8 |
| *Tibouchina longifolia* | | shrub | NAT | NA | NA | |  | 11 |
| *Tibouchina mollis* | | shrub | NAT | NA | NA | |  | 30 |
| *Tibouchina ochypetala* | | treelet | NAT | NA | NA | |  | 5 |
| *Tournefortia bicolor* | | shrub | NAT | NA | NA | |  | 7 |
| *Tournefortia fuliginosa* | | treelet | NAT | NA | NA | |  | 26 |
| *Tournefortia glabra* | | treelet | NAT | NA | NA | |  | 1 |
| *Trifolium dubium* | | herb | IN | Europe | Paleartic | | Temperate | 15 |
| *Trifolium pratense* | | herb | IN | Eurasia, north Africa | Paleartic | | Temperate | 3 |
| *Trifolium repens* | | herb | IN | Europe, east Asia | Paleartic | | Temperate | 192 |
| *Triolena pustulata* | | herb | E | NA | NA | |  | 5 |
| *Trisetum spicatum* | | herb | NAT | NA | NA | |  | 27 |
| *Tropaeolum adpressum* | | liana | NAT | NA | NA | |  | 15 |
| *Uncinia hamata* | | herb | NAT | NA | NA | |  | 3 |
| *Uncinia paludosa* | | herb | NAT | NA | NA | |  | 18 |
| *Uncinia phleoides* | | herb | NAT | NA | NA | |  | 11 |
| *Urera baccifera* | | shrub | NAT | NA | NA | |  | 1 |
| *Urera caracasana* | | shrub | NAT | NA | NA | |  | 3 |
| *Urtica leptophylla* | | herb | NAT | NA | NA | |  | 3 |
| *Valeriana microphylla* | | shrub | NAT | NA | NA | |  | 18 |
| *Valeriana pilosa* | | herb | NAT | NA | NA | |  | 4 |
| *Valeriana plantaginea* | | herb | NAT | NA | NA | |  | 1 |
| *Vallea stipularis* | | shrub | NAT | NA | NA | |  | 1 |
| *Vasconcellea microcarpa* | | shrub | NAT | NA | NA | |  | 1 |
| *Verbena litoralis* | | herb | NAT | NA | NA | |  | 54 |
| *Verbesina arborea* | | shrub | NAT | NA | NA | |  | 14 |
| *Vernonanthura patens* | | treelet | NAT | NA | NA | |  | 7 |
| *Veronica persica* | | herb | IN | Eurasia | Paleartic | | Temperate | 2 |
| *Veronica serpyllifolia* | | herb | NAT | NA | NA | |  | 33 |
| *Viburnum hallii* | | treelet | NAT | NA | NA | |  | 6 |
| *Viburnum toronis* | | tree | NAT | NA | NA | |  | 4 |
| *Vicia andicola* | | herb | NAT | NA | NA | |  | 5 |
| *Viola dombeyana* | | herb | NAT | NA | NA | |  | 20 |
| *Viola stipularis* | | herb | NAT | NA | NA | |  | 14 |
| *Vismia baccifera* | | treelet | NAT | NA | NA | |  | 12 |
| *Vismia sprucei* | | treelet | NAT | NA | NA | |  | 1 |
| *Vulpia bromoides* | | herb | IN | Eurasia, north Africa | Paleartic | | Temperate | 3 |
| *Vulpia myuros* | | herb | IN | Mediterranean | Paleartic | | Temperate | 18 |
| *Wedelia grandiflora* | | shrub | NAT | NA | NA | |  | 13 |
| *Weinmannia balbisiana* | | treelet | NAT | NA | NA | |  | 7 |
| *Weinmannia lentiscifolia* | | tree | NAT | NA | NA | |  | 2 |
| *Weinmannia magnifolia* | | tree | NAT | NA | NA | |  | 3 |
| *Werneria nubigena* | | herb | NAT | NA | NA | |  | 6 |
| *Werneria pumila* | | herb | NAT | NA | NA | |  | 22 |
| *Xanthosoma daguense* | | herb | NAT | NA | NA | |  | 1 |
| *Xenophyllum humile* | | herb | NAT | NA | NA | |  | 4 |
| **Status** | Biogeographic current condition of the species in Ecuador | | | | |  | | |
| **Biogeographic origin** | Origin place of the species | | | | |  | | |
| **Phytogeographic region** | Phytogeographic region to which the place of origin of the species belongs | | | | |  | | |
| **Latitudinal area** | Zone of origin | | | | |  | | |
| **IN** | Introduced species | | | | |  | | |
| **NAT** | Native species | | | | |  | | |
| **E** | Endemic species | | | | |  | | |
